# Regulation of the First Committed Step in Lipopolysaccharide Biosynthesis Catalyzed by LpxC Requires the Essential Protein LapC (YejM) and HslVU Protease

**DOI:** 10.3390/ijms21239088

**Published:** 2020-11-29

**Authors:** Daria Biernacka, Patrycja Gorzelak, Gracjana Klein, Satish Raina

**Affiliations:** Unit of Bacterial Genetics, Gdansk University of Technology, 80-233 Gdansk, Poland; darbiern@student.pg.edu.pl (D.B.); patrycja.gorzelak@gmail.com (P.G.)

**Keywords:** lipopolysaccharide, LapB, LapC, YejM, LpxC, HslV/U protease, FabZ, RpoE

## Abstract

We previously showed that lipopolysaccharide (LPS) assembly requires the essential LapB protein to regulate FtsH-mediated proteolysis of LpxC protein that catalyzes the first committed step in the LPS synthesis. To further understand the essential function of LapB and its role in LpxC turnover, multicopy suppressors of Δ*lapB* revealed that overproduction of HslV protease subunit prevents its lethality by proteolytic degradation of LpxC, providing the first alternative pathway of LpxC degradation. Isolation and characterization of an extragenic suppressor mutation that prevents lethality of Δ*lapB* by restoration of normal LPS synthesis identified a frame-shift mutation after 377 aa in the essential gene designated *lapC*, suggesting LapB and LapC act antagonistically. The same *lapC* gene was identified during selection for mutations that induce transcription from LPS defects-responsive *rpoE*P3 promoter, confer sensitivity to LpxC inhibitor CHIR090 and a temperature-sensitive phenotype. Suppressors of *lapC* mutants that restored growth at elevated temperatures mapped to *lapA*/*lapB*, *lpxC* and *ftsH* genes. Such suppressor mutations restored normal levels of LPS and prevented proteolysis of LpxC in *lapC* mutants. Interestingly, a *lapC* deletion could be constructed in strains either overproducing LpxC or in the absence of LapB, revealing that FtsH, LapB and LapC together regulate LPS synthesis by controlling LpxC amounts.

## 1. Introduction

The cytoplasm of Gram-negative bacteria, such as *Escherichia coli*, is surrounded by an inner membrane (IM), which is a phospholipid bilayer that separates an aqueous periplasmic compartment containing a thin layer of peptidoglycan from the outer membrane (OM). The OM of Gram-negative bacteria is an asymmetric bilayer in nature with phospholipids located in its inner leaflet, while the lipopolysaccharide (LPS) is located on the outer leaflet [1]. LPS is a complex glycolipid, essential for the bacterial viability and constitutes the major amphiphilic component of OM in most of the Gram-negative bacteria, including *E*. *coli* [1,2]. However, LPS is highly heterogeneous in the composition and comprised of a mixture of different glycoforms, whose synthesis and accumulation are controlled by various regulatory factors and growth conditions [3,4,5,6,7]. The preferential synthesis of certain glycoforms that determine the LPS composition involves the induction or activation of regulatory factors that include the main envelope stress-responsive regulator RpoE, two-component systems such as BasS/R, PhoP/Q and Rcs in addition to regulatory sRNAs [5,7,8,9]. Despite the heterogeneity in the composition of LPS, they in general share domains composed of a membrane-anchored bisphosphorylated and acylated β(1→6)-linked GlcN disaccharide, termed lipid A, to which a carbohydrate moiety of varying size is attached [10,11,12]. The latter may be divided into a proximal core oligosaccharide and, in smooth-type bacteria, a distal *O*-antigen. The inner core usually contains residue(s) of 3-deoxy-α-d-*manno*-oct-2-ulosonic acid (Kdo) and L-*glycero*-D-*manno-*heptose (L,D-Hep). The lipid A part and the inner core are generally conserved in the structure, but often have nonstoichiometric modifications [2,5,8,11].

LPS biosynthesis begins with the acylation of UDP-GlcNAc by LpxA with *R*-3-hydroxymyristate derived from *R*-3-hydroxymyristoyl-ACP, which is also used by FabZ for the phospholipid synthesis. Thus, *R*-3-hydroxymyristoyl-ACP serves as a common precursor for the synthesis of phospholipids and LPS [3,13,14]. The second reaction of the lipid A biosynthesis is catalyzed by LpxC deacetylase, constituting the first committed step in the LPS synthesis, as the equilibrium constant for the first reaction catalyzed by LpxA is unfavorable due to reversible nature of reaction. The biosynthesis of LPS in *E*. *coli* proceeds in a discontinuous manner. First, the tetraacylated lipid A (lipid IV_A_) precursor is synthesized, which requires six essential enzymes. After the synthesis of lipid IV_A_ precursor, the transfer of two Kdo residues constitutes an additional essential step in the LPS biosynthesis [3,9,15,16]. This Kdo addition ensures the incorporation of secondary lauroyl and myristoyl chains resulting in the synthesis of hexaacylated lipid A [11,16]. Following the synthesis of Kdo_2_-lipid A, further sugars are sequentially transferred by membrane-associated glycosyltransferases for the completion of core biosynthesis. Thus, Kdo_2_-lipid A constitutes the minimal LPS structure that is required for the viability of bacteria like *E. coli* under standard laboratory growth conditions [10,11]. However, viable strains synthesizing LPS composed of only glycosylation-free lipid IV_A_ precursor can be constructed, which can be propagated only under slow growth conditions at low temperatures with a narrow growth range around 23 °C, due to poor recognition by MsbA LPS flippase [11]. Furthermore, when the bacterial LPS synthesis is compromised at different steps of its biosynthesis, such as when the LPS is composed of minimal structures like Kdo_2_-lipid A Δ*waaC*, Kdo_2_-lipid IV_A_ or with only lipid IV_A_ precursor, such mutant bacteria exhibit severe permeability defects and the highly elevated RpoE-dependent envelope stress response [11]. Similarly, when the LPS assembly is compromised due to an imbalance in the synthesis of LPS and phospholipid as in Δ*lapB* strains, they too exhibit the super elevation in the RpoE-dependent stress response [17]. These LPS defects are sensed via the activation of Rcs two-component system that causes an induction of *rpoE* mRNA synthesis from its *rpoE*P3 promoter [18].

**Figure 1:**
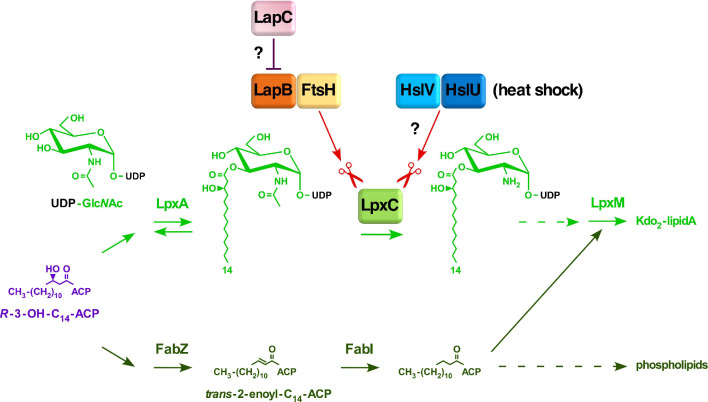
Model of regulation of balanced LPS and phospholipid biosynthesis: *R*-3-hydroxymyristoyl-ACP serves as a common metabolic precursor for the LpxC–dependent LPS biosynthesis and the FabZ-mediated phospholipid biosynthesis. LpxC stability is regulated by previously established FtsH/LapB and, from this study, via the negative regulation by LapC counteracting the LapB/FtsH pathway and also at high temperature by HslVU proteolysis of LpxC.

It is well established that the synthesis of LPS and phospholipids is tightly co-regulated and held at a nearly constant ratio of 0.15 to 1.0 [19]. Any alterations in the balance of phospholipid and LPS are not tolerated by bacteria and cause cell death. Thus, either the excess or reduction in the LPS synthesis in comparison to the phospholipid synthesis causes the lethality [17,20]. This balance is achieved by tightly regulated turnover of unstable LpxC enzyme to maintain the flux of common precursor *R*-3-hydroxymyristoyl-ACP for the utilization in either LPS or phospholipid biosynthesis. Thus, the key players in this pathway are FabZ and LpxC enzymes. The *fabZ* gene encodes the *R*-3-hydroxymyristoyl-ACP dehydratase, catalyzing the first step in the phospholipid biosynthesis [15]. Hence, the utilization of the fundamental common metabolic substrate of LpxC and FabZ, *R*-3-hydroxymyristoyl-ACP, constitutes an essential branch point in the biosynthesis of phospholipids and the lipid A part of LPS (Figure 1).

The turnover of LpxC enzyme is catalyzed by the essential IM-located, Zn-dependent metalloprotease FtsH in concert with another essential IM-anchored LPS assembly heat shock protein LapB (Figure 1) [17,20,21]. Although FtsH has several substrates, the essentiality of FtsH protease stems from its key role in proteolysis of LpxC to prevent excess build up in the LPS biosynthesis at the expense of synthesis of phospholipids [22]. The notion that FtsH and LapB work together to regulate LpxC proteolysis is based on biochemical data revealing their co-purification and the increased accumulation of LpxC in either *ftsH* or *lapB* mutants [17]. Thus, mutations in either the *ftsH* gene or the *lapB* gene result in the increased abundance of LpxC, which leads to excess of LPS over phospholipids due to depletion of the common precursor *R*-3-hydroxymyristoyl-ACP [17,20,23]. Consistent with their role in regulating LpxC amounts, a deletion of either *ftsH* or *lapB* are tolerated in strains carrying either a hyperactive allele of the *fabZ* gene *sfhC21* or when the LPS synthesis is dampened due to mutations in either *lpxA* or *lpxD* genes [17,23].

In addition, the lethal phenotype of Δ*lapB* or Δ(*lapA lapB*) can also be overcome, if either the LPS synthesis is impaired due to mutations in the gene encoding heptosyltransferase I (*waaC*) or when non-coding sRNA SlrA (MicL), a translational repressor of the most abundant Lpp protein, is overproduced [17]. Interestingly, *lapA* and *lapB* genes are co-transcribed from three promoters: the distal promoter located upstream of the *pgpB* gene, the middle promoter recognized by the heat shock sigma factor RpoH and the last one resembling house-keeping promoters [17]. Such a transcriptional organization suggests coupling of transcription of *lapA*/*B* genes with phospholipid metabolism using *pgpB* co-transcription. The *pgpB* gene encodes phosphatidylglycerophosphatase, an enzyme that is a part of the pathway of phosphatidylglycerol biosynthesis, and hence associated with phospholipid metabolism [24].

Although we showed that LapA and LapB proteins interact with FtsH and co-purify with LPS, it is unclear how LapB senses LPS to regulate LpxC proteolysis. Based on modelling prediction and subsequent crystal structure studies, it was shown that the LapB protein contains in its N-terminal domain nine tetratricopeptide repeats (TPR) and a C-terminal rubredoxin-like domain, both of which were found to be essential for its function and together provide a scaffold for the assembly and recruitment of LpxC and various LPS biosynthetic proteins [17,25].

It has been often reported that some unstable proteins can be substrates of multiple proteases. For example, the RpoH heat shock sigma factor is a substrate for FtsH and also the heat shock-inducible HslVU (ClpQY) protease [26,27,28]. Similarly, unstable SulA and RcsA proteins can be subjected to degradation by either Lon or HslVU proteases [29,30]. Thus, in this study, experiments were undertaken to further understand the molecular basis of the essentiality of LapB, address if additional interacting partners of LapB exist and if there is the possibility of alternative mechanism of LpxC proteolysis. We rationalized that if LapB is interacting with the additional protein(s), the deletion of such a gene could be lethal. However, point mutations in such a gene should exhibit the elevated transcriptional response of RpoE sigma factor quite like that observed with *lapB* mutants or in strains synthesizing the severely truncated LPS [11,17]. In parallel, a multicopy suppressor approach was utilized, asking if overexpression of any gene can rescue the lethal phenotype of either Δ*lapB* or Δ(*lapA lapB*) strains. Thus, in the first approach, Tn*10*-linked point mutations that simultaneously exhibited the elevated *rpoE*P3 promoter activity, the sensitivity to the LpxC inhibitor CHIR090 to different extents and membrane permeability defects were analyzed leading to the identification of three point mutations: one having a frame-shift mutation resulting in truncation after 377 amino acid residue isolated three times, the second mutation introduces a stop codon after amino acid residue 190, another resulting into a single amino acid F349S substitution, in the essential *yejM* gene. During the completion of this work, YejM has been speculated to either maintain lipid homeostasis or be involved in the transport of cardiolipin or exhibit a metal-dependent phosphatase activity [31,32,33,34,35,36,37]. We designated this gene *lapC* based on identified genetic and biochemical interactions of LapC with LapB. Mutations in the *lapC* gene exhibited a nearly 2 to 8-fold increased expression of *rpoE*P3-*lacZ* fusion, with the inability to grow on rich medium above 37 °C, the sensitivity to the CHIR090 LpxC inhibitor and highly decreased amounts of LpxC and LPS. Suppressor analysis identified several mutations mapping to the *lapA*/*B* operon, *ftsH* and *lpxC* genes. In the second approach, we show that overexpression of *hslV* encoding the protease subunit can bypass the lethality of a Δ*lapB* derivative. Overexpression of *hslV* alone or *hslVU* was found to repress the elevated accumulation of LpxC in Δ*lapB* mutants, which was validated by pulse-chase experiments revealing an alternative pathway of LpxC degradation.

## 2. Results

### 2.1. Rationale

**Figure 2:**
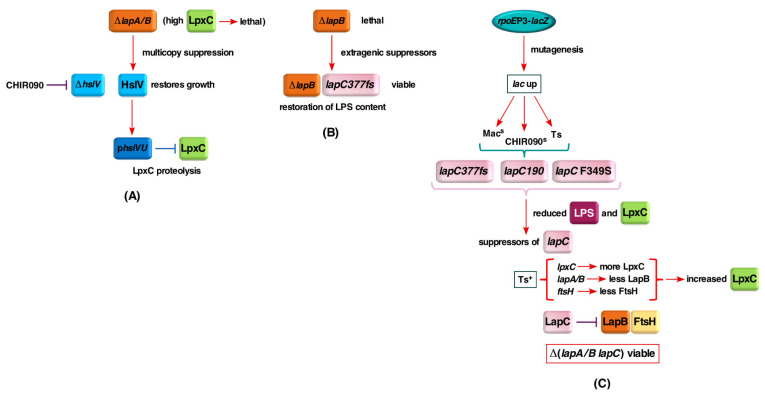
Schematic drawing illustrating three major approaches that identified additional players in the regulation of LpxC proteolysis. The multicopy suppressor approach led to discovery of the HslVU-dependent proteolysis of LpxC in the absence of LapB (**A**). In search of suppressors that bypass the lethality of a *lapB* deletion, the *lapC377fs* mutation was isolated that restored the normal LPS synthesis and the viability in the absence of LapB (**B**). In the third approach, based on mutagenesis, CHIR090- and temperature-sensitive mutants with defects in LPS identified the *lapC* gene. Suppressors of *lapC* mutants revealed that LapC works together with LapB/FtsH to regulate LpxC proteolysis and the essential *lapC* gene is dispensable in the absence of LapB (**C**).

It is well established that LpxC constitutes the first rate-limiting step in the LPS biosynthesis and its amounts are tightly regulated by its proteolysis by the FtsH/LapB complex. However, it remains unknown, how LapB exerts its effect. Based on several lines of experimental evidence, LapB was proposed to act as a scaffold in the IM, where LPS biosynthetic enzymes, involved in lipid A and LPS core biosynthesis, are recruited [17]. During this process, LapB could monitor the accumulation of LPS in the IM and direct LpxC to proteolysis via FtsH to prevent unwanted excess of LPS biosynthesis. Consistent with this presumption, we have shown co-purification of LapB with LPS and FtsH [17]. However, it is unknown how LPS amounts are sensed and if FtsH is the sole protease that regulates LpxC turnover. Thus, we took several approaches: in one case, we sought to identify if additional proteases are involved in the degradation of LpxC using the multicopy suppressor approach to identify additional factors that can bypass the lethality of Δ*lapB* or Δ*lapA/B* mutants (Figure 2A). This multicopy suppressor approach identified the HslUV protease complex to participate in the turnover of LpxC and its overexpression circumvented the essentiality of LapB. Isolation of extragenic suppressors of strains lacking LapB, that restored normal LPS biosynthesis and bypassed LapB essentiality, identified a unique frame-shift mutation in the essential gene *yejM* causing truncation of its periplasmic domain and was designated *lapC* (Figure 2B). In a complementary approach, we sought temperature-sensitive point mutations that render *E*. *coli* sensitive to the LpxC inhibitor CHIR090, exhibit permeability defects (sensitivity to growth on MacConkey agar) and simultaneously induce transcription from the *rpoE*P3 promoter (Figure 2C). CHIR090 is a known inhibitor of LpxC and severe defects in LPS biosynthesis cause permeability defects and turn on transcription of the gene encoding the alternative sigma factor RpoE, which responds to defects in LPS and outer membrane protein maturation [11,17,18,38,39]. This approach further led to the isolation of several point mutations in the *lapC* gene establishing an essential role in the LPS assembly and sensing. LapC was shown to genetically and physically interact with LapB and found to control LpxC levels together with FtsH based on the identification of extragenic suppressors of *lapC* loss-of-function mutations.

### 2.2. Overexpression of Heat Shock Protein HslV Protease Can Bypass the Lethality Due to the Deletion of the lapB Gene, Whose Product Is Required for the Regulated Assembly of LPS

We previously showed that the *lapB* gene is essential for the viability of *E*. *coli* and its deletion can be very poorly tolerated only in few strain backgrounds at 30 °C on minimal medium unless an extragenic suppressor is present [17]. The essential function of LapB was shown to be due its role in LPS biosynthesis by regulating turnover of LpxC in concert with the essential Zn-dependent metalloprotease encoded by the *ftsH* gene. FtsH has been shown to be required for degradation of LpxC [17,20,21]. In this work, we sought to isolate factor(s) that bypass the lethality of Δ*lapB* mutants in order to identify, if any alternative back up proteolytic pathway exists in *E*. *coli* by using a multicopy suppressor approach. Thus, a complete library of all cloned ORFs, where genes are expressed from the IPTG-inducible P_T5_-*lac* promoter from ASKA collection in the vector pCA24N [40], was introduced into the Δ(*lapA lapB*) strain SR17187 and plated on LA medium at 42 °C in the presence of 75 μM IPTG as an inducer for the gene expression. Plasmid DNA was isolated from such transformants and used to retransform the parental Δ(*lapA lapB*) strain to confirm the plasmid-mediated suppression. Such plasmid DNAs were sequenced and their analysis revealed that besides previously identified multicopy suppressors a set of plasmids contained the *hslV* gene, which encodes the catalytic subunit of ATP-dependent protease HslUV [27,41].

The *hslV* gene is co-transcribed as a part of heat shock-inducible operon *hslVU* also called *clpQY* [27,42]. HslVU comprises the prokaryotic counterpart of eukaryotic proteasome [43,44]. HslV exhibits homology to the β-subunits of the eukaryotic 20S proteosome, while HslU shows the high amino acid sequence homology to the ClpX protease [27]. HslV on its own exhibits the proteolytic activity, which is significantly enhanced by HslU in the presence of ATP [27,41]. We previously identified HslVU (ClpQY) on the basis of its overexpression repressing abnormally induced the heat shock gene expression or that induced by the addition of puromycin [27,45] and was indeed subsequently found to degrade the heat shock sigma factor RpoH [26].

Thus, we performed sets of controlled transductions, using bacteriophage P1 grown on Δ*lapA/B* and Δ*lapB* strains, into wild-type strain BW25113 derivatives transformed with either the vector alone or by the plasmid carrying either the *hslV* gene alone or with cloned *hslVU* genes in the presence of 75 μM IPTG. Results from such transductions convincingly demonstrate that the essential *lapB* gene can be deleted, when the *hslV* gene alone is overexpressed (Table 1). Furthermore, the transduction frequency was enhanced by approximately 3-fold, when *hslVU* genes were co-overexpressed (Table 1). It should be noted that Δ*lapB* transductants appear at a low frequency and are obtained after the prolonged incubation with small sized colonies, when the vector alone is present (Table 1). Taken together, we can conclude that the *lapB* gene becomes nonessential, when the *hslV* gene is overexpressed.

### 2.3. Overexpression of Either hslV or hslUV Rescue the Lethality of LapB Deletion by Enhancing LpxC Degradation in a LapB-Independent Manner

**Figure 3:**
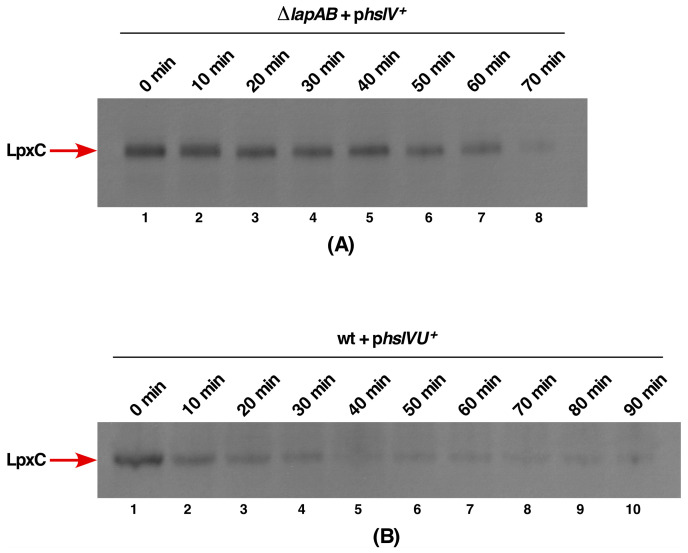
Overexpression of either the *hslV* gene alone or *hslVU* genes results in enhanced proteolysis of LpxC. (**A**) Cultures of Δ*lapA/B* strain carrying the inducible *hslV* gene on a plasmid were grown in M9 medium, adjusted to an OD_595_ of 0.2 in LB medium. Expression of the *hslV* gene was induced by the addition of 75 μM IPTG at 42 °C. An equivalent amount of total proteins from indicated time points were resolved by SDS-PAGE and LpxC levels were determined by immunoblot analysis using LpxC-specific antibodies, revealing a gradual decrease of LpxC. (**B**) Expression of *hslVU* genes from the pET24b vector in BL21 strain was induced by the addition of 75 μM IPTG at 30 °C for 15 min at an OD_595_ of 0.1. Cultures were washed and shifted to 42 °C and further host transcription stopped by the addition of rifampicin. LpxC levels were determined using the equivalent amount of total proteins by immunoblot analysis using LpxC-specific antibodies.

To address the molecular basis of suppression of the lethality of either the *lapB* or *lapA*/*B* deletion by HslV or HslVU overproduction, we rationalized that the unstable LpxC could be a substrate for the HslV protease. We have earlier shown that LpxC is stabilized in the absence of LapB and its levels are enhanced in a Δ*lapA/B* derivative that leads to the increased synthesis of LPS and a consequent bacterial lethality. Thus, we measured levels of LpxC in a Δ*lapA/B* derivative transformed by the plasmid carrying the *hslV* gene expressed from a tightly regulated inducible P_T5_-*lac* promoter using Western blotting technique. The expression of the *hslV* gene in the strain lacking *lapA*/*B* genes was induced by the addition of 75 μM IPTG and an equivalent amount of culture was withdrawn after 10 min intervals and proteins resolved by SDS-PAGE. Western blot analysis with anti-LpxC antibodies revealed a progressive reduction in amounts of LpxC, with less than 50% of LpxC present after 20 min incubation (Figure 3A, lane 3). After the prolonged incubation of 70 min, a barely detectable level of LpxC was observed (Figure 3A, lane 8). It should be noted that in the absence of LapB, LpxC is stable and its levels are elevated at all conditions [17]. Results from these experiments showed that LpxC can be subjected to proteolysis in the absence of LapB, which is known to work with FtsH, establishing an alternative mechanism of control of LpxC levels and helps to explain the identification of the *hslV* gene as a suppressor of lethality of Δ*lapB*.

As HslV is known to exhibit a weak peptidase activity, which is enhanced by HslU, we examined the impact of co-overexpression of *hslVU* genes in the wild-type background and analyzed the fate of LpxC in pulse chase experiments. A plasmid expressing both genes from the T7 promoter in pET24b was used for such studies. For these studies, the expression of *hslUV* genes was induced for 15 min and further transcription stopped by the addition of 200 μg/mL of rifampicin and incubated at 42 °C during chase. The equivalent amount of aliquots were collected at indicates intervals. Total proteins were resolved on SDS-PAGE and analyzed by the Western blotting technique, using LpxC-specific antibodies (Figure 3B).

As can be seen, LpxC amounts rapidly decline during the chase time, confirming that LpxC is a substrate for the HslUV protease that does not require FtsH protease (Figure 3B), since the complete host transcription was shut down by the addition of rifampicin. Following the 10 min chase itself, approximately only 20% of LpxC species that cross-react with anti-LpxC antibodies are observed (Figure 3B, lane 2). After 40 min, only the background level of LpxC can be detected (Figure 3B, lane 5).

Thus, these results provide a convincing evidence that HslV can degrade LpxC in the absence of LapB/FtsH and this degradation is enhanced, when HslU is also present providing a rationale explanation for the identification of HslV as a multicopy suppressor of the *lapB* deletion and preventing the lethal accumulation of LpxC.

### 2.4. hslV and hslU Mutants Exhibit the Sensitivity to the LpxC Inhibitor CHIR090

**Figure 4:**
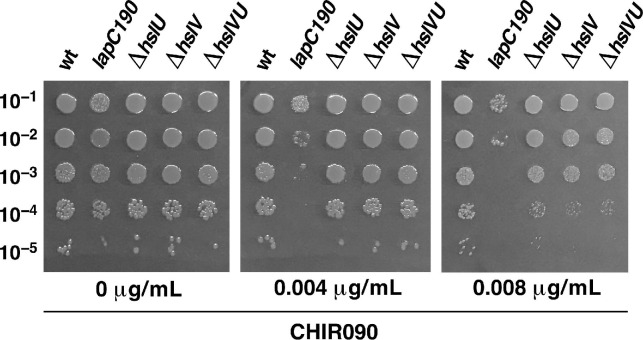
The absence of *hslV* and *hslVU* genes confers the sensitivity to the LpxC inhibitor CHIR090. Exponentially grown cultures of the wild type and its isogenic derivatives lacking genes encoding HslVU protease subunits were adjusted to an OD_595_ of 0.1 and serially spot diluted at 30 °C on LA agar supplemented with or without varying concentrations of CHIR090 as indicated. As a control, the isogenic CHIR090-sensitive strain with the *lapC190* mutation was included. Data presented are from one of the representative experiments.

It is well-established that the LpxC activity can be inhibited by the CHIR090 inhibitor, which binds near the Zn-binding domain of LpxC that determines its active site [39]. Mutations that confer resistance to CHIR090 are known to map to the *lpxC* gene [38]. Thus, we wondered if deletion derivatives of *hslV* and *hslU* genes exhibit any altered sensitivity to sub-lethal doses of CHIR090. Isogenic strains of wild type BW25113 and its derivatives lacking HslUV protease subunits were spot tested on LA agar with and without supplementation of CHIR090 at a concentration that is not deleterious to the wild-type strain. These experiments revealed that the deletion derivative of the *hslV* gene or a strain lacking both subunits exhibit a 100-fold reduction in the viability, when challenged with the sub-lethal concentration of CHIR090, and also a reduction in the colony size (Figure 4). Under the same conditions, Δ*hslU* mutants, although more sensitive than the parental strain, exhibit only a moderate sensitivity to CHIR090 (Figure 4). It should be however noted that the lack of HslUV does not confer the extreme sensitivity as observed with a *lapC* mutant expressing only its IM anchor (Figure 4 and see further below). Thus, we can conclude that consistent with a role for HslUV in the turnover of LpxC, removal of either both subunits of this protease complex or HslV catalytic subunit enhances the sensitivity to CHIR090, which is a specific inhibitor of LpxC.

### 2.5. An Extragenic Suppressor Mutation in the lapC Gene Restores the Normal LPS Synthesis and Prevents the Lethality in the Absence of LapB

In our earlier work, we reported that a *lapB* deletion could not be tolerated in *E. coli* W3110 strain unless a suppressor mutation was present [17]. To gain further understanding of LapB function, additional extragenic suppressors were sought by transducing Δ*lapB* in W3110 that allowed a deletion of the *lapB* gene. A novel mutation that allowed deletion of either the *lapB* gene or *lapA/B* genes was identified resulting into construction of SR8348 (Table S1). Marking of suppressor mutation and further transduction of this suppressor mutation was found to allow growth of Δ*lapA/B* strain also in BW25113 on LA medium at 30 °C and thus was not the strain-specific suppressor mutation. Complementation and DNA sequence analysis revealed that this suppressor mutation is due to a frame-shift mutation in the *yejM* essential gene [31,46] and based on further genetic interactions designated the *lapC* gene. This mutation causes an insertion of C residue at nt position 1133 resulting into frame-shift after 377 amino acid (*lapC377fs*), with the addition of 13 aa residues after Thr377, leading into the truncation of C-terminal periplasmic domain. LapC (YejM) structures have been described recently revealing five IM-spanning helices in the N-terminus and a large C-terminal globular domain connected by a linker domain [32,34,47,48].

**Figure 5:**
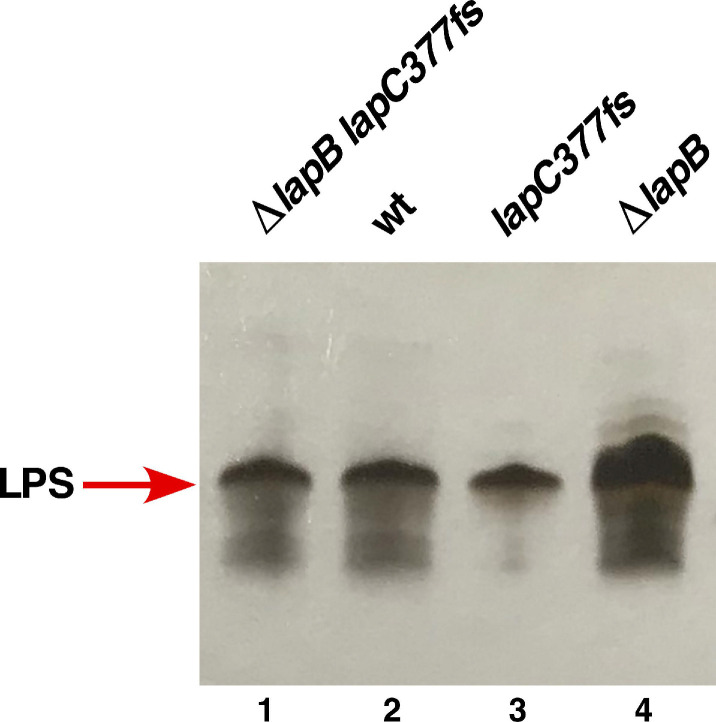
The *lapC377fs* mutation restores the wild-type LPS content in Δ*lapB*. Equivalent amounts of whole cell lysate treated with Proteinase K were resolved on a 14% SDS-Tricine gel and LPS revealed by silver staining. The arrow indicates the position of LPS and the relevant genotype of strains is depicted.

**Figure 6:**
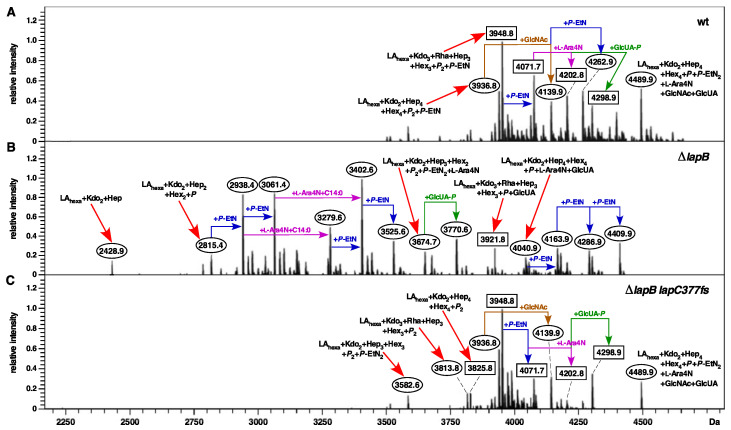
The *lapC377fs* mutation suppresses the accumulation of LPS precursors and restores the normal LPS synthesis in Δ*lapB*. Charge-deconvoluted mass spectra in the negative ion mode of LPS from the wild type (**A**), its Δ*lapB* (**B**) and Δ*lapB lapC377fs* (**C**) derivatives. Cultures were grown in phosphate-limiting medium at 30 °C. Mass numbers refer to monoisotopic peaks. Mass peaks with rectangular boxes correspond to the glycoform containing the third Kdo. Ovals—derivatives with two Kdo residues with either complete or incomplete core.

To address the molecular basis of suppression by the *lapC377fs* mutation, LPS of the wild type, its Δ*lapB*, *lapC377fs* and (Δ*lapB lapC377fs*) derivatives was extracted from whole cell lysates treated with Proteinase K. Equivalent amount of extracts were resolved on a SDS-Tricine gel and LPS was revealed after silver staining. Comparison of LPS profile revealed an excess of LPS in Δ*lapB* and a reduced LPS content in *lapC377fs*, which is restored to wild-type levels in (Δ*lapB lapC377fs*) (Figure 5). Next, we used purified LPS and analyzed them by mass spectrometry. Consistent with previous results [17], spectra of Δ*lapB* derivative reveal that LPS is composed of mixture of complete and its precursor derivatives. This is evident from the presence of several prominent mass peaks (with mass ranging from 2428.9 to 3770.7 Da) that correspond to early intermediates of LPS as compared to their absence in the spectra of wild type strain (Figure 6). Some of these mass peaks representing early intermediates include the mass peak at 2815.4 Da in the LPS of Δ*lapB* with the predicted composition of LA_penta_ + Kdo_2_ + Hep_2_ + Hex_2_ + *P*. Of significance is the absence of early intermediates of LPS and the restoration of accumulation of LPS with the complete core with hexaacylated lipid A in (Δ*lapB lapC377fs*) derivative (Figure 6C). Mass peaks of LPS of wild type and (Δ*lapB lapC377fs*) derivative, representing glycoforms with either 2 Kdo or 3 Kdo residues, are present in both spectra with *P*-EtN and L-Ara4N non-stochiometric modifications. The predicted composition of main mass peaks is marked (Figure 6). Thus, mass peaks at 3936.8 and its derivatives with mass peaks at 4139.9 and 4489.9 Da, corresponding to the glycoform with 2 Kdo residues with different additional non-stoichiometric substitutions, are present in spectra of the wild type and (Δ*lapB lapC377fs*). Furthermore, mass peaks corresponding to glycoforms IV and V derivatives with a third Kdo + Rhamnose (mass peaks such as 3948.8, 4071.7, 4202.8 and 4298.9 Da) are prominent in the spectra of wild type and (Δ*lapB lapC377fs*), but absent in Δ*lapB* spectra, reflecting the suppression of Δ*lapB* LPS defect in the presence of *lapC377fs* suppressor mutation. Restoration of normal LPS composition in the Δ*lapB lapC377fs* derivative suggests that the truncation within the C-terminal domain of LapC could prevent the excessive synthesis of LPS in a Δ*lapB* background, which is consistent with the reduced LPS synthesis in *lapC* mutants (see below).

### 2.6. The C-Terminal Truncation of the Periplasmic Globular Domain in the Essential LapC Protein Results in the Induction of RpoE-Dependent Envelope Stress Response, a Temperature-Sensitive Phenotype and the Sensitivity to the LpxC Inhibitor CHIR090

**Figure 7:**
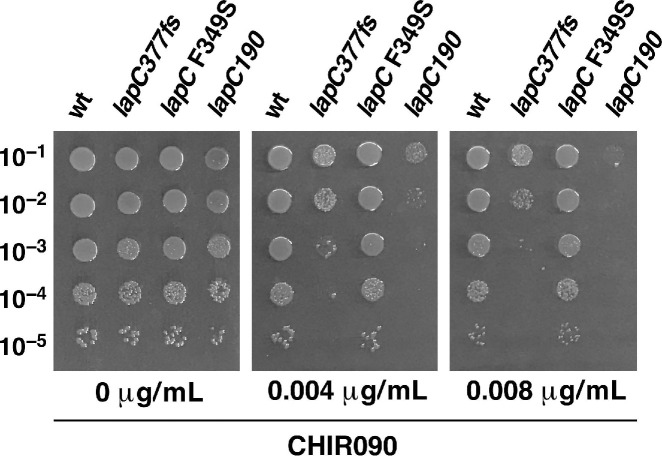
Truncation of the C-terminal periplasmic domain of LapC confers the sensitivity to the LpxC inhibitor CHIR090. Exponentially grown cultures of the wild type and its isogenic derivatives with point mutations in the *lapC* gene were adjusted to an OD_595_ of 0.1 and serially spot diluted at 30 °C on LA agar supplemented with or without varying concentrations of CHIR090 as indicated.

In complementary studies, we sought to identify additional partners that could sense either the accumulation of LPS in the IM or defects in LPS biosynthesis and regulate LpxC amounts. As the major players identified thus far are essential for bacterial growth such as either the *lapB* gene or the *ftsH* gene, we reasoned that any previously unidentified partner protein could also be essential for the bacterial viability. To achieve this goal, temperature-sensitive (Ts) Tn*10*-linked point mutations that conferred the increased activity of single-copy *rpoE*P3-*lacZ* promoter were isolated. This promoter was chosen as its activity is specifically induced, when LPS biosynthesis or assembly is severely compromised [18]. Among such mutants, only those isolates that were sensitive to the CHIR090 inhibitor of LpxC and unable to propagate on MacConkey agar were retained (Figure 2 and Figure 7). Mapping and complementation analysis of Tn*10*-linked mutations identified four independent Ts mutants with permeability defects defined by SR19041, SR19750, SR22861 and SR22862 with a mutation linked > than 95% to the *bcr* gene located at 49 min. Subcloning and further complementation approach led to the cloning of the *lapC* gene, which reversed the Ts phenotype, restored growth on MacConkey agar plates as well as restored the nearly wild-type activity of the *rpoE*P3-*lacZ* fusion. Next, we PCR amplified the *lapC* gene from the chromosomal DNA of four strains and their DNA sequence analysis revealed: SR19041 (*lapC190*) has a stop codon TGA replacing TGG (Trp190), SR19750 and SR22861 have a frame-shift *lapC377fs* mutation and SR22862 (*lapC* F349S) contains a single amino acid change resulting into 349F to 349S change (TTT to TCT). It is of interest, as described above (Section 2.5), that the *lapC377fs* mutation was independently identified as a suppressor mutation of the Δ*lapB* derivative.

**Figure 8:**
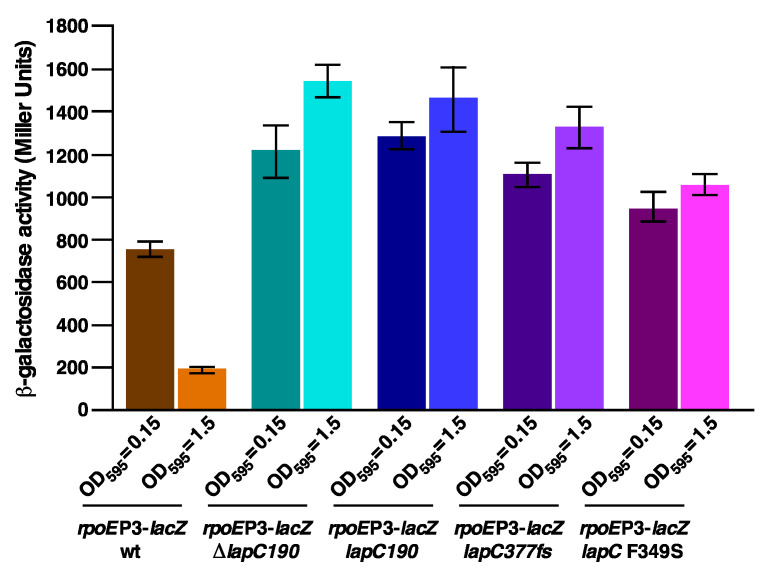
Mutations that cause truncation of periplasmic domain of LapC induce transcription from the *rpoE*P3 promoter, which specifically responds to severe defects in LPS. Exponentially grown isogenic strains of the wild type and its derivatives with various *lapC* mutations as indicated, carrying the single-copy chromosomal *rpoE*P3-*lacZ* fusion, were analyzed for the *β*-galactosidase activity. Cultures were adjusted to an OD_595_ of 0.05 and allowed to grow in LB medium at 30 °C. Aliquots of samples were drawn after different time intervals and used to measure the *β*-galactosidase activity. Error bars represent a S.E. of three independent measurements.

Among three Ts mutations, *lapC190* expresses only the IM anchor of LapC, comprising of five predicted TM helices but lacks the entire periplasmic domain. This mutation is identical to a mutation described in the strain LH530 exhibiting membrane permeability defects [31,46]. However, the most frequent mutation identified is *lapC377fs*. The F349 amino acid residue is highly conserved and located in the Mg^++^ ion-binding pocket in the pseudo-hydrolase domain and in the putative phosphatase active site [32,34]. Comparison of sensitivity of three isogenic *lapC* mutant derivatives to CHIR090 revealed that *lapC190* is extremely sensitive to this LpxC inhibitor, followed by the *lapC*377*fs* derivatives at the concentration, when growth of the parental strain is not affected (Figure 7, lanes 1, 3&4). However, the *lapC* F349S derivative showed only a small decrease in the colony size upon the exposure to CHIR090 and is consistent with a weaker induction of the *rpoE*P3-*lacZ* promoter fusion (Figure 8). Taken together, these results establish that LapC is required for the regulation of LpxC, since *lapC190* and *lapC377fs* mutations confer the hypersensitivity to the CHIR090 inhibitor and were further followed. To prevent read through of stop codon in the original *lapC190* derivative, for further comparative analysis and verification of results, we used a Δ*lapC190* derivative (GK6075) constructed by recombineering that expresses only the N-terminal 190 amino acids defining the IM anchor and the periplasmic domain is replaced by an excisable antibiotic cassette.

Measurement of *β*-galactosidase activity of *lapC* mutant bacteria revealed a 2 to 8-fold increase in the *rpoE*P3-*lacZ* activity as compared to the wild type depending upon the growth phase and the mutation in the *lapC* gene (Figure 8). As SR19041 (*lapC190*) and GK6075 (with the antibiotic cassette inserted in the coding region after the 190th amino acid) expresses only the intact IM N-terminal anchor region devoid of the C-terminal domain, both of these derivatives were used in this assay for comparison (Figure 8). Most striking results reveal a 6 to 8-fold induction of the *rpoE*P3-*lacZ* fusion in either *lapC190* or Δ*lapC190* and *lapC377fs* derivatives, when grown in the stationary phase (Figure 8). All of these derivatives are unable to propagate on MacConkey agar with a Ts phenotype. These results correlate very well with the sensitivity to the CHIR090 LpxC inhibitor of strains carrying either *lapC190* or *lapC377fs* mutations with a lower impact of the *lapC* F349S mutation. Thus, the induction of transcription from the *rpoE*P3 promoter that responds to LPS defects accompanied by the Ts phenotype, the sensitivity to the CHIR090 inhibitor and membrane permeability defects allows us to conclude that LapC is required for the cell envelope integrity and the regulation of LPS biosynthesis as illustrated by the suppression of lethality and the restoration of wild-type LPS content in Δ*lapB* by *lapC* loss-of-function mutations.

### 2.7. The lapC190 Mutation Resulting in the C-Terminal Truncation Causes the Reduction in Amounts of LpxC Leading to the Reduced Accumulation of LPS

**Figure 9:**
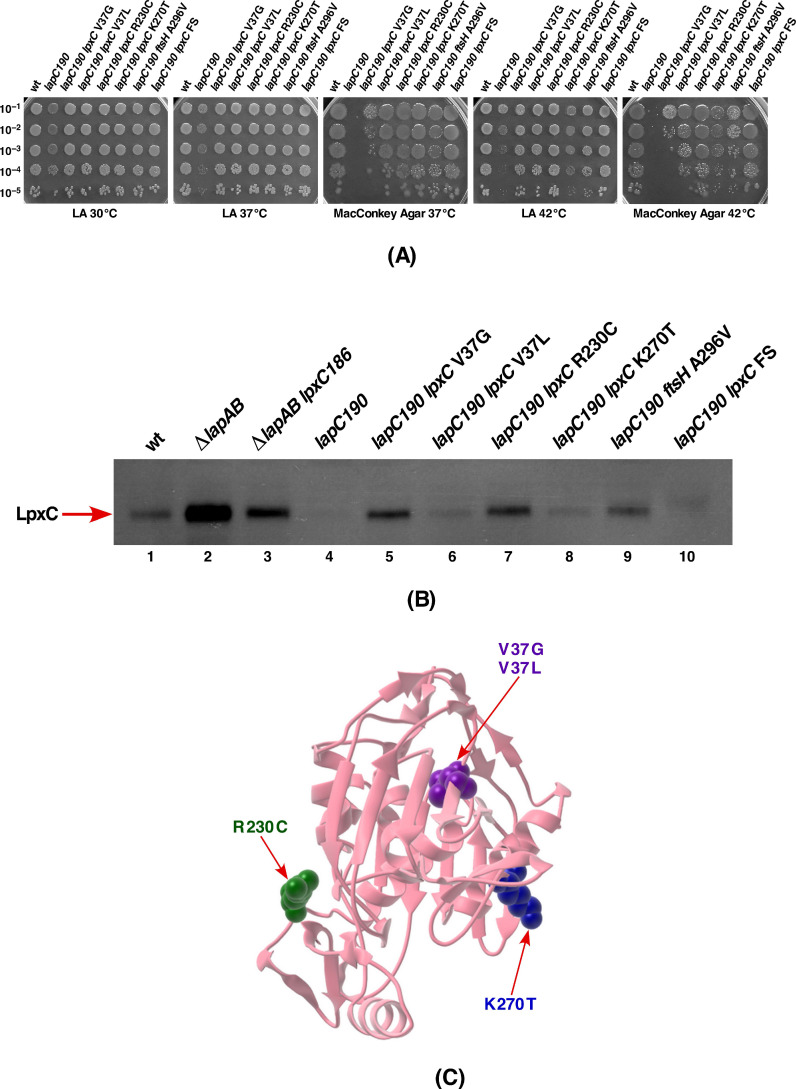
*lapC190* mutant bacteria exhibit a reduction in LPS amounts and the temperature-sensitivity (Ts) phenotype. (**A**) Exponentially grown cultures with indicated genotypes were adjusted to an OD_595_ of 0.1 and spot diluted on LA and MacConkey agar at different temperatures. (**B**) Exponentially grown cultures of the wild type, its *lapC190* derivative and strains with various suppressor mutations mapping to the *lpxC* gene were grown under permissive growth conditions. Equivalent amounts of total cellular proteins were resolved by SDS-PAGE and proteins transferred by Western blotting and subjected to immunoblotting, using LpxC-specific antibodies. (**C**) The position of amino acids residues in the structure of LpxC (PDB 4MQY, [49]), whose specific alterations suppress the Ts phenotype of a *lapC190* mutation and cause elevation of LpxC levels.

As *lapC* mutant bacteria exhibit the extreme sensitivity to the sub-lethal concentration of the LpxC inhibitor CHIR090, have the elevated induction of *rpoE*P3 promoter that is activated in the response to severe defects in LPS and restore the LPS composition of a Δ*lapB* derivative, suggest that LapC has a specific role in the LPS assembly/biosynthesis. To further gain information about the function of LapC, firstly growth on LA and MacConkey agar was measured. Quantification of bacterial growth revealed that mutations in the C-terminal part of the *lapC* gene confer the temperature-sensitive phenotype (Figure 9A). However, this Ts phenotype and the membrane permeability defect reflected by the sensitivity to MacConkey agar can be reversed by various suppressor mutations (see below). 

Next, we analyzed if LpxC amounts are affected and what is the status of LPS amounts. Since among three *lapC* mutations identified in this work a *lapC190* mutation confers a more stringent phenotype as reflected by the sensitivity to CHIR090, we measured levels of LpxC from cellular extracts obtained from exponentially grown cultures of the isogenic wild type, its *lapC190* derivative and as controls Δ*lapA*/*B* and a strain carrying a suppressor mutation of *lapA/B* deletion mapping to the *lpxC* gene (Δ*lapA/B lpxC186*). Cultures for such experiments were grown under permissive growth conditions of M9 minimal medium at 30 °C and cell extracts analyzed by immunoblotting. Results from such a representative experiment reveal barely detectable levels of LpxC as compared to the isogenic parental strain in *lapC190* bacteria (Figure 9B, lanes 1&4, respectively). These results of highly diminished amounts of LpxC in the *lapC190* mutant are in sharp contrast to elevated LpxC levels in a Δ*lapA*/*B* derivative and its partial reduction in the *lpxC186* derivative of *lapAB* mutation (Figure 9B, lanes 2&3).

**Figure 10:**
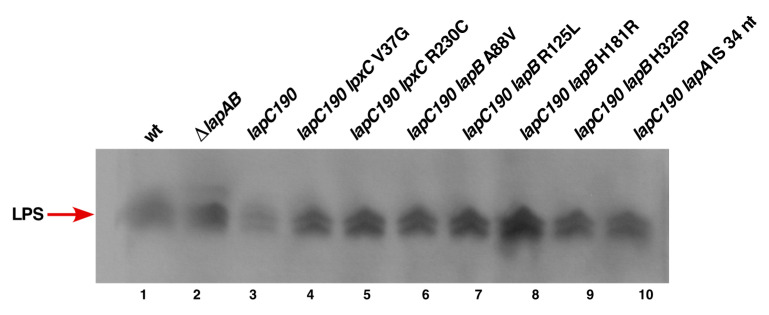
LPS levels are highly reduced in *lapC* mutants lacking the C-terminal periplasmic domain, which are restored by various suppressor mutations mapping to either *lpxC*, *lapA* or *lapB* genes. Isogenic bacterial cultures of the wild type and its derivatives with the indicated genotype were grown up to an OD_595_ of 0.5 at 30 °C. An equivalent portion of whole cell lysates were applied to a 14% SDS-Tricine gel and transferred by Western blotting. Relative amounts of LPS were revealed by immunoblotting with the LPS-specific monoclonal antibody WN1 222-5 using chemiluminescence detection kit.

To correlate the reduction in LpxC amounts in *lapC190* mutants with the LPS accumulation, we directly measured LPS amounts using Proteinase K-treated whole cell lysates as described previously [11]. Samples were resolved on a SDS-Tricine gel and LPS was transferred by Western blotting. Amounts of LPS were revealed using a LPS-specific WN1 222-5 monoclonal antibody [50]. Results from immunoblots clearly show that the amount of LPS that cross reacted with WN1 222-5 monoclonal antibody in the *lapC190* mutant was significantly reduced as compared to that observed with the wild type (Figure 10, lanes 1&3), establishing a role for LapC in regulating LpxC amount and determining amount of LPS accumulation in line with results of restoration of LPS composition in a (Δ*lapB lapC377fs*) derivative (Figure 6).

### 2.8. Extragenic Suppressors That Restore LPS Levels in lapC190 Mutants Map to lpxC, lapA, lapB and ftsH

To further gain insights into the function of LapC, we took the advantage of Ts phenotype and the inability to grow on MacConkey agar of the *lapC190* and *lapC377fs* derivatives. Thus, we sought extragenic suppressors that either restored growth on LA agar at 42 °C or allowed growth on MacConkey agar by plating cultures under non-permissive growth conditions. Several spontaneous suppressors were isolated and 29 of them were marked with transposon Tn*10* as described earlier [17]. To map the suppressor mutation, linked Tn*10* insertion positions were obtained from recombinant cosmid clones by directly sequencing the Tn*10* insertion position. Out of 29 suppressors, 26 suppressor mutations could be grouped into three complementation groups (Table 2). Ten suppressors were found to be linked to *zab*::Tn*10*, 15 were linked to *pyrF*::Tn*10* and one was found to be linked to *greA*::Tn*10*. These map positions suggest that suppressor mutations linked to *zab*::Tn*10* could have a mutation in the *lpxC* gene, those linked to *pyrF*::Tn*10* mapping to the *lapA/B* locus and the last one linked to the *greA* gene could be having a mutation in the *ftsH* gene. Thus, we isolated chromosomal DNA from all 26 suppressor-containing mutants and directly sequenced the chromosomal DNA region spanning the coding and their flanking regions of *lpxC*, *lapA*, *lapB* and *ftsH* genes.

The results obtained from this DNA sequence analysis are presented in Table 2. Four independently isolated suppressor strains that restored growth at 42 °C on LA medium and also allowed growth to some extent on MacConkey agar contained a single amino acid exchange of V37G in the coding region of the *lpxC* gene (Figure 9A, Table 2). However, the strain with LpxC V37G suppressor grows poorly on MacConkey agar as compared to the LpxC V37L suppressor-containing strain in the *lapC190* background (Figure 9A). Similarly, three suppressor mutations that restored growth of either *lapC190* or *lpxC377fs* mutant strains had an exchange of R230 to C residue in the coding region of the *lpxC* gene (Figure 9A, Table 2). Sequence analysis of another suppressor-containing strain GK6094 revealed a frame-shift mutation due to the deletion of 2 nt in the stop codon that results in the extension of LpxC wild-type sequence by 20 amino acids. The C-terminal domain of LpxC is known to comprise the recognition domain for its proteolysis by FtsH protease [51]. Thus, this extension of LpxC by the unusual addition of 20 unrelated amino acids might prevent FtsH-mediated proteolysis, resulting into its stabilization and was verified by the examination of LpxC levels (see below).

Two other strains SR22738 and GK6078 with the suppressor mutation in the *lpxC* gene have single amino acid alterations resulting into V37L and K270T changes, respectively (Table 2, Figure 9C). The isolation of five independent isolates that lead to the substitution of Val37 residue is consistent with recent identification of LpxC V37G as a stable variant in *Klebsiella pneumoniae* [52]. Thus, we speculate that suppressor mutations that we identified in the *lpxC* gene render the encoding protein resistant to proteolysis and was investigated further.

### 2.9. Suppressors of lapC190 Mutation Mapping to the lpxC Gene Prevent Its Degradation and Lead to the Restoration of LPS Amounts

As LpxC and LPS amounts are limiting in strains carrying *lapC* mutations, we reasoned that suppressors mapping to the *lpxC* gene could act by stabilizing LpxC and restore LPS amounts to the wild-type levels. Thus, LpxC amounts were analyzed by Western blotting of cell extracts obtained from such suppressor-containing strains. As controls, the equivalent amount of total proteins from the parental wild type and its isogenic derivatives carrying either a Δ*lapA/B* mutation, the strain with the *lpxC186* suppressor mutation of *lapB* deletion and the derivative with the *lapC190* mutation were analyzed (Figure 9B). The most striking observation from the immunoblot analysis is the stabilization of LpxC in strains that carry the suppressor mutation in the *lpxC* gene. These results can explain the molecular basis of suppression and establish that LpxC is destabilized in *lapC* mutant derivatives with truncation of the periplasmic domain. All strains with *lpxC* suppressors accumulate more LpxC than even the wild type and the original *lapC190* mutant. The most prominent among them are strains with LpxC V37G, LpxC R230C and with a frame shift resulting into extension of LpxC by 20 amino acids, with a pronounced increase in the amounts of LpxC (Figure 9B). The Δ*lapA/B* strain, that served as a control, indeed accumulates lot more LpxC (Figure 9B, lane 2). Taken together, these results allow us to conclude that truncation of the C-terminal periplasmic domain destabilizes LpxC and suppressors mapping to the *lpxC* gene that restore growth at high temperature render LpxC more stable to balance the LPS synthesis.

To validate above results in the context of LPS synthesis and establish a role of LpxC degradation in the *lapC* mutant derivative and its stabilization in strains with the suppressor mutation, LPS amounts were analyzed by immunoblotting using the LPS-specific antibody. Whole cell lysates from two most prominent suppressor-containing strains with LpxC V37G and LpxC R230C mutations were analyzed in such comparative experiments. Results from such a representative experiment reveal the restoration of LPS synthesis as compared to the highly reduced LPS content in the *lapC190* derivative (Figure 10, lanes 4&5 vs. lane 3). These results provide a rationale explanation of decreased LPS synthesis due to destabilization of LpxC, when LapC function is impaired, and the restoration of LPS synthesis in the presence of suppressors mapping to the *lpxC* gene, which render LpxC more stable.

### 2.10. A Range of Suppressors of lapC190 Map to the lapA/lapB Operon That Could Either Disturb LapB Protein Structure or Dampen Its Abundance by Preventing Its Synthesis

**Figure 11:**
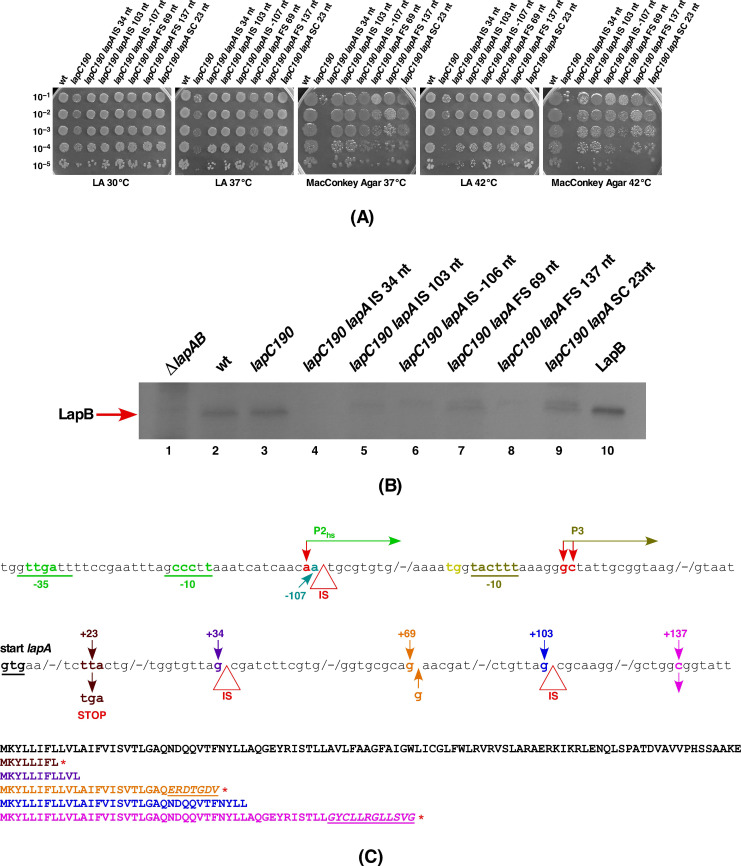
Suppressors of *lapC190* that restore growth at high temperature mapping either to the promoter region of the *lapA/B* operon or in the *lapA* gene reduce the LapB abundance. (**A**) Isogenic cultures of wild type, its *lapC190* derivative and *lapC190* with a specific suppressor mutation in the *lapA* gene or its promoter region were adjusted to an OD_595_ of 0.1, serially diluted, 5 μL aliquots spotted on either LA agar or MacConkey agar at various indicated temperatures and plates incubated for 24 h. (**B**) Immunoblots of whole cell lysates obtained from isogenic strains with indicated genotypes using LapB-specific antibodies. An equivalent amount of total proteins was loaded. As controls, extracts from Δ*lapA*/*B* and purified LapB (lanes 1&10) were applied. (**C**) The position of various suppressor mutations in the *lapA* gene is indicated. The presence of IS element in either the coding region or the promoter region is indicated by a triangle at the specific nt position. Other mutations causing frame shifts or introducing stop codons, resulting into either truncation of LapA or alterations in the amino acid sequence, are indicated and underlined with the indicated stop codon as * symbol.

The LapB protein is essential for bacterial growth due to its role in mediating LpxC degradation by either activating FtsH to recognize LpxC or its presentation to degradation pathway by acting as a scaffold for proteins involved in the LPS assembly/biosynthesis [17]. Consistent with this notion, we earlier demonstrated that LpxC is stabilized in the absence of LapB that leads to excess of build up LPS and causes the bacterial lethality [17]. Suppressors mapping to the *lpxC* gene, which reduce the LpxC synthesis or its accumulation, can allow a deletion of the *lapB* gene [17]. However, phenotypes of *lapC* mutants suggest an antagonistic action of LapC with LapB, as loss-of-function mutations have the opposite phenotype (less LPS and reduced LpxC stability) and (Δ*lapB lapC377fs*) combination restores the normal LPS synthesis. Thus, the isolation of suppressors of the *lapC190* and *lapC377fs* mutations mapping to the *lapA/B* operon should shed further light on how LapB and LapC operate. DNA sequence analysis revealed that 15 out of 26 suppressors had a mutation either in the coding region of the *lapB* structural gene or insertion sequence element (IS) in the *lapA* gene or in the promoter region of *lapA/B* operon, or a stop codon within the *lapA* gene (Table 2, Figure 11). Two additional mutations caused frame shifts in the *lapA* gene, disrupting the co-translation of downstream essential *lapB* gene (Figure 11C). It should be noted that *lapA* and *lapB* genes are transcribed as an operon and their translation is coupled [17]. The isolation of a number of IS elements in the *lapA* gene suggests that due to polarity such insertions could abrogate transcription of the *lapB* gene, thus prevent proteolysis of LpxC in the absence of LapB (Figure 11).

To validate the isolation of suppressor mutations in the *lapA*/*B* operon that restore bacterial growth at high temperature of *lapC190* and *lapC377fs* mutant derivatives, we quantified the degree of suppression at various levels. Examination of growth of *lapC190* derivatives with suppressor mutations in either the *lapA* gene or in the promoter region of the *lapA/B* operon by spot dilution of isogenic cultures at different temperatures revealed restoration of growth of *lapC190* derivatives on LA medium at 37 and 42 °C (Figure 11A). Such suppressor-carrying strains also exhibited restoration of growth on MacConkey agar, which is very restrictive for a strain with a *lapC190* mutation (Figure 11A).

To address the molecular basis of suppression either due to the frame-shift mutation(s) or the presence of IS element in the *lapA* gene, we directly examined the comparative abundance of LapB in cell extracts of such suppressor-containing strains. To measure levels of LapB, the equivalent amount of total proteins from whole cell lysates of strains with various class of mutations in the *lapA* structural gene or its promoter region were examined by immunoblotting with anti-LapB antibodies (Figure 11B). As a control, we used the equivalent amount of total proteins from the isogenic Δ*lapA/B*, the wild type and *lapC190* derivatives. Purified LapB was used as a control to validate the cross-reactivity of anti-LapB antibodies. Examination of such an immunoblot showed that the presence of either IS element, or a frame-shift mutation in the *lapA* gene or the IS element after one of the major promoters of *lapA/B* operon either abolished the LapB synthesis or severely reduced its synthesis as compared to either the wild type or the *lapC190* derivative (Figure 11B,C). These results support the conclusions that transcription and translation of *lapA* and *lapB* mRNAs are coupled and suppressor mutations that restore growth of *lapC* mutant strains mapping to the *lapA* gene act by blocking the LapB synthesis. Thus, the IS element and frame-shift mutations in the *lapA* gene and promoter would as well result in reduced transcription of the *lapB* gene, which can cause an increased accumulation of LpxC and hence suppress *lapC* mutants, which have less LPS and less LpxC.

### 2.11. Suppressor Mutations in the Coding Region of the lapB Gene Map to Conserved Amino Acids in TPR Motifs that Result in the Reduced Abundance of LapB and Restoration of the LPS Synthesis

**Figure 12:**
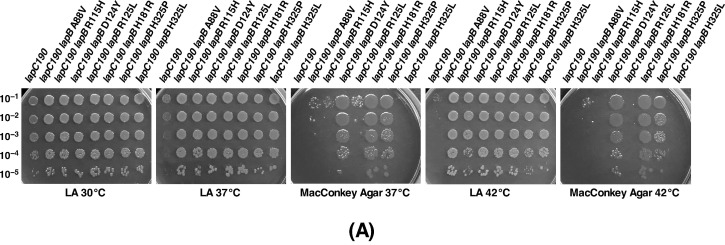

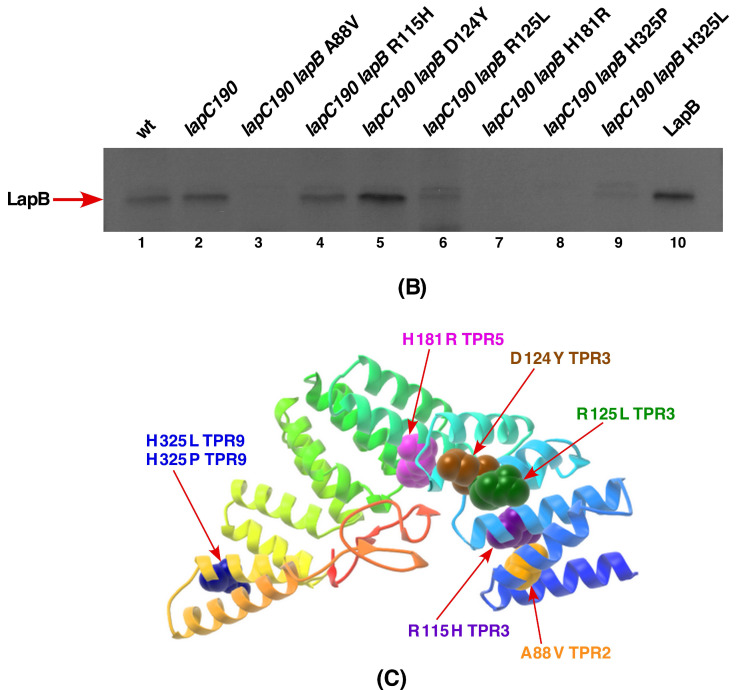
Suppressors mapping to the *lapB* gene that restore growth of *lapC* mutant bacteria reduce the LapB abundance. (**A**) Growth of isogenic cultures of strains with *lapC190* and with suppressor mutations in the *lapB* gene was quantified by spot dilution on LA and MacConkey agar at various temperatures. The genotype and incubation temperature are indicated. (**B**) Immunoblot of total cellular proteins from various strains, used in growth measurement, with LapB-specific antibodies. Purified LapB (lane 10) serves as a control to validate the cross-reactivity of LapB and its position on immunoblot. Equivalent amount of proteins were resolved by SDS-PAGE prior to immunoblotting. (**C**) The position of various mutations in LapB with the indicated relevant TPR motif are shown in the structure of LapB (PDB 4ZLH, [25]).

DNA sequence analysis revealed that eight suppressor-containing strains have single amino acid substitutions resulting into A88V, R115H, D124Y, R125L, H181R, H325L and H325P exchanges. All these amino acids in the *lapB*-coding sequence are highly conserved residues in tetratricopeptide repeats (TPR) elements (Table 2, Figure 12). Thus, the mutation of these residues could prevent interaction with proteins such as FtsH or interaction with the rubredoxin domain [17,25]. For example, the side-chain mutation H181R might result in the weaker rubredoxin-TPR binding (Figure 12).

Next, we systematically quantified the degree of suppression of *lapC190* mutation by above mentioned substitutions in the *lapB* gene by the spot dilution assay of strains with the suppressor mutation as compared to its parental strain. Consistent with their selection at 42 °C, all *lapB* substitutions restored growth of the parental *lapC190* strain on LA medium at 37 and 42 °C (Figure 12A). However, growth on MacConkey agar, reflecting the suppression of permeability defects, was restored only with suppressor D124Y, H181R and H325P mutations.

To further investigate how various substitutions impact the accumulation of LapB was addressed by Western blotting of total cellular proteins with anti-LapB antibodies. Examination of immunoblots revealed that most of mutations mapping to the *lapB* gene cause a severe reduction in the amounts of LapB as compared to its levels in the wild-type strain, with the exception of LapB D124Y substitution (Figure 12B). It is likely that D124Y substitution could cause the conformational change being located in the conserved TPR3 motif (Figure 12C). Consistent with this argument are the results of growth restoration of the *lapC190* mutation on LA as well as on MacConkey agar in the presence of D124Y substitution. Thus, we can conclude that amino acid substitutions in the *lapB* gene result in the loss of function or perturb folding of LapB protein, which could render it proteolytically sensitive and prevent its accumulation (Figure 12B).

As LapB amounts determine amounts of the LPS synthesis and loss of LapB is lethal due to the increased synthesis of LPS causing depletion of pools of the *R*-3-hydroxymyristoyl-ACP precursor for both phospholipid and LPS biosynthesis, we measured the accumulation of LPS in strains with suppressor mutations in either *lapA* or *lapB* genes as compared to the *lapC190* derivative. Measurement of LPS by immunoblots using the WN1 222-5 monoclonal antibody in parallel with strains with suppressor mutations in the *lpxC* gene clearly show that the synthesis of LPS is restored in all investigated variants of either LapA or LapB (Figure 10, lanes 6 to 10). These results provide the molecular basis of suppression of *lapC* derivative lacking the C-terminal periplasmic domain, as suppressor mutations exhibit the increased accumulation of LPS as compared to the highly reduced LPS content in *lapC* mutants. These results again support the isolation of another *lapC377fs* suppressor mutation that bypasses the lethality of Δ*lapB* derivatives (Figure 6). Thus, these results allow us to conclude that LapC and LapB jointly regulate the LPS synthesis by acting in an antagonistic manner.

### 2.12. Suppressor Mutation in the FtsH Gene Maps to the Conserved Region Required for Its ATPase Activity

**Figure 13:**
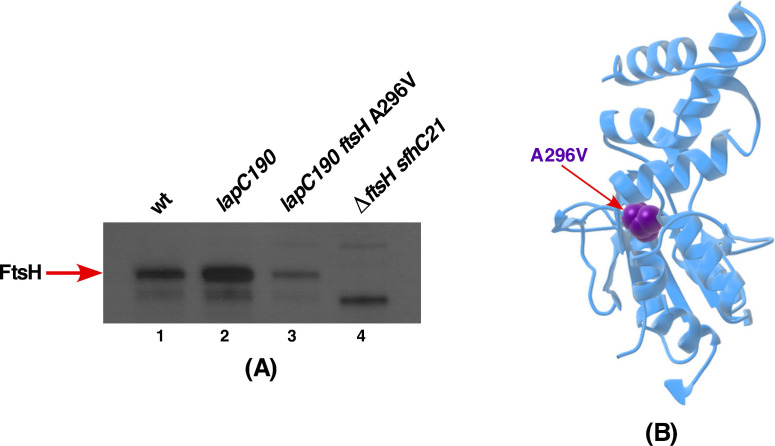
FtsH A296V suppressor mutation causes the reduction in FtsH levels. (**A**) Immunoblot analysis of total cellular extracts obtained from the wild type, its derivatives with *lapC190, lapC190 ftsH* A296V and as the negative control isogenic strain with Δ*ftsH sfhC21* mutation. An equivalent amount of proteins was resolved by SDS-PAGE and immunoblots were treated with a FtsH-specific antibody. Arrow indicates the position of FtsH. (**B**) The position of FtsH amino acid residue 296 on its crystal structure (PDB 1LV7 [54]) is shown.

Lastly, one of remaining suppressor mutation was found to map to the *ftsH* gene, which encodes the protease responsible for the LpxC degradation. This suppressor mutation causes exchange of conserved Ala296 by Val in the Second Region of Homology (SRH) domain (Figure 13). Mutations in the SRH domain are often associated with the loss of ATPase activity, which is critical for its proteolytic activity [53]. Consistent with its location in the SRH domain, *lapC190 ftsH* A296V derivative was found to accumulate more LpxC (Figure 9).

As LapB and FtsH jointly regulate the LPS synthesis by regulating LpxC turnover, we examined levels of FtsH in the strain with *lapC190 ftsH* A296V using total cellular extracts. Western blot analysis revealed that FtsH levels are significantly reduced in the *lapC190 ftsH* A296V derivative as compared to the isogenic parental strain carrying the *lapC190* mutation (Figure 13A,B). As a negative control, total cell extracts from the Δ*ftsH* strain that is viable due to the *sfhC21* mutation, which encodes a hyperactive FabZ variant and lacks any cross-reacting FtsH were applied (Figure 13A). Thus, we can conclude that FtsH A296V in *lapC190* has the reduced amount of FtsH and due to its location in the ATPase-regulating domain could act by preventing LpxC degradation and restore the LPS synthesis.

### 2.13. The lapB Gene Is Dispenable in lapC Loss-of-Function Mutations and Overexpression of lpxC Can Bypass the Essentiality of the lapC Gene

Results presented in above sections revealed a reduced content of LPS in strains with either the *lapC190* or *lapC377fs* mutation and restoration of the LPS synthesis with either suppressor mutations mapping to the *lapA/B* operon or viability of Δ*lapB lapC377fs* derivatives prompted us to address in more detail if indeed the essential *lapB* gene is dispensable when LapC is dysfunctional. In line with such arguments, the suppressor mutation mapping to the *lpxC* gene render LpxC variants resistant to proteolysis and hence exhibit the increased accumulation of LpxC. Thus, we undertook genetic experiments using bacteriophage P1-mediated transductions to introduce either a Δ*lapA*/*B* or Δ*lapB* variants in either the *lapC190* or the *lapC377fs* backgrounds.

Results from transductions (Table 3) revealed that Δ*lapB* or Δ*lapA/B* can be readily introduced in strains carrying loss-of-function mutation in the *lapC* gene namely *lapC190* and *lapC377fs* as compared to the extremely low frequency of transduction with the parental BW25113 strain as a recipient. Furthermore, transductants in the wild-type strain form very small colonies and are obtained after the prolonged incubation. It should be noted that the *lapB* gene is essential in most of wild-type strains, however its deletion is tolerated poorly on M9 minimal medium at 30 °C and such strains grow very poorly [17] unless extragenic suppressor mutation(s) is present. These results are also consistent with the isolation of *lapC377fs* as an extragenic suppressor of Δ*lapB* (Figure 6) with the restoration of normal LPS synthesis. These results allow us to conclude that LapB is non-essential, when LapC is dysfunctional.

As LpxC amounts are limiting in *lapC* mutants, with the same rationale as presented above, we performed transductions to introduce the Δ*lapC* mutation in the wild-type background transformed with a plasmid expressing the *lpxC* gene under the inducible P*_T5_-lac* promoter. Firstly, the Δ*lapC* derivative, which served as a donor in P1 transductions, was constructed in a background where the Δ*lapC* derivative is maintained viable due to the expression of the *lapC* gene in a low-copy cosmid clone. Using controlled experiments, a Δ*lapC* mutation could be introduced when the *lpxC* gene is mildly induced by the addition of 50 μM IPTG (Table 3) but not in the absence of IPTG with supplementation of glucose (0.2%) when the *lpxC* expression is repressed. These results are consistent with the isolation of suppressor mutations of *lapC* mutants that accumulate more LpxC. Thus, we can conclude that LapC and LapB act antagonistically to maintain the balanced synthesis of LpxC and prevent its uncontrolled degradation and deletion of the *lapC* gene can be tolerated when either the *lpxC* gene is mildly overexpressed or in the absence of LapB.

### 2.14. Co-Purification of LapB and LapC

**Figure 14:**
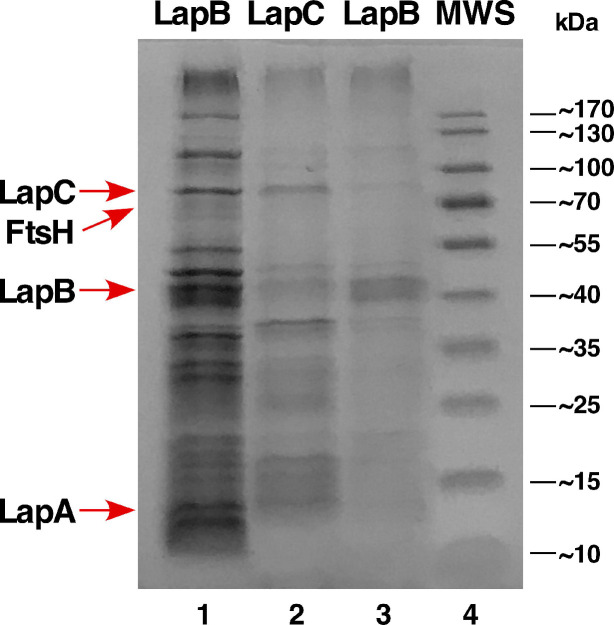
LapC and LapB show physical interaction. Purification profile of proteins from IM fractions after 100 μM and 500 μM IPTG addition to induce *lapA* and *lapB* transcription. Lanes 1 and 3 indicate co-purification of His-tagged LapA with LapB and LapC from IM fractions of total proteins. Lane 2 depicts elution profile of His_6_-LapC and its co-elution with LapB. Proteins were resolved on a 12% SDS-PAGE. The identity of LapC, LapB, LapA and FtsH are shown by arrows.

Given the genetic data presented in this work, all data point to the evidence of genetic interaction between the *lapB* and *lapC* genes. Thus, we performed pull-down experiments using the N-terminally His_10_-tagged LapA. For this overexpression, different inducer concentrations of either 100 μM or 500 μM IPTG were used. Here, the plasmid used carries both *lapA* and *lapB* genes and hence are co-expressed. Purified IM fractions were subjected to Ni-NTA chromatography and eluted proteins were identified by MALDI-TOF. Consistent with LapA and LapB co-purification, we also identified LapC as one of the proteins that could be part of this complex (Figure 14, lanes 1 and 3). In parallel, we could also pull-down LapB protein, when IM fractions from LapC induction were used for affinity purification (Figure 14, lane 2). Thus, co-purification of LapB and LapC provide the additional evidence of interaction of these proteins, supporting data of genetic interaction.

### 2.15. The Expression of the lapC Gene Is Induced at High Temperatures

**Figure 15:**
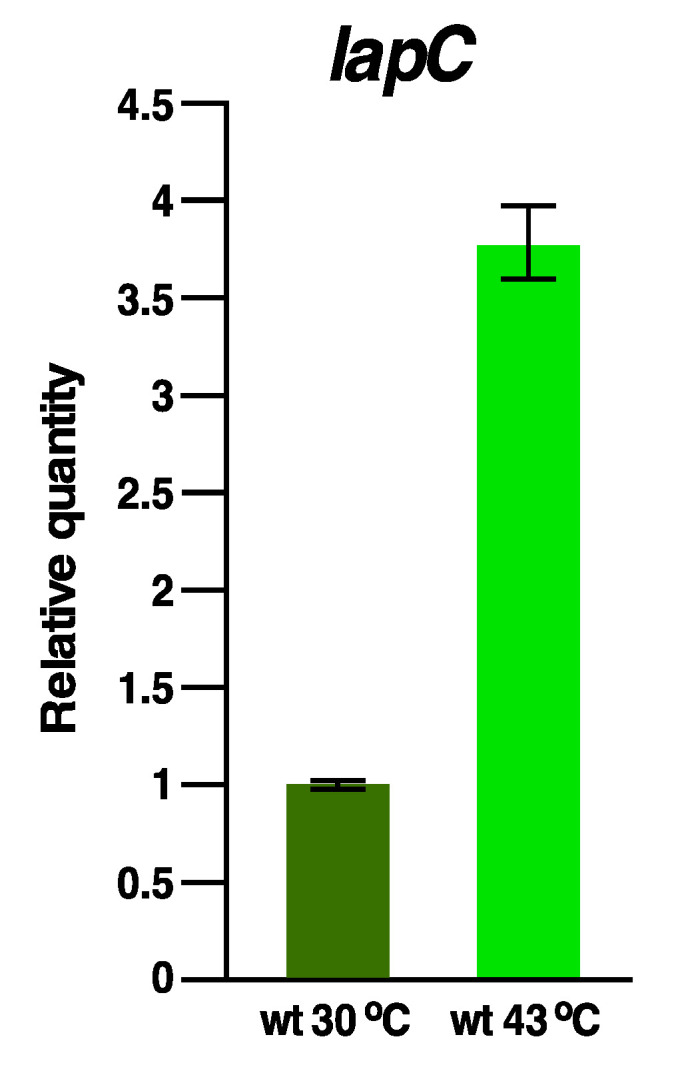
Transcription of the *lapC* gene is induced upon a shift to high temperature. qRT-PCR analysis of mRNA isolated from wild-type bacteria grown up to an OD_595_ of 0.2 in M9 minimal medium either at 30 °C or after a 15-min shift to 42 °C. Data presented are from RNA isolated from three biological replicates and error bars are indicated.

Although LapC is essential under all growth conditions, the synthesis of several genes is known to vary under stress conditions. For example, transcription of the *lapA/B* operon is heat shock inducible. As LapB and LapC interact, we further addressed if transcription of *lapC* is altered by a shift to high temperature. The relative abundance of *lapC* transcripts was measured by qRT-PCR using total RNA extracted from wild-type bacteria grown either at 30 °C or after a transient 15-min shift to 42 °C. Quantification of gene expression pattern revealed a significant 3.5-fold increase in the abundance of *lapC* transcripts after 15-min exposure to heat shock conditions (Figure 15). 

Thus, *lapC* transcription is not only sustained at high temperature but is rather induced, consistent with its essentiality under all growth conditions. However, how the *lapC* transcription is induced at high temperature remains unknown (see Discussion).

## 3. Discussion

It is well established that amounts of LpxC, which catalyzes the first rate-limiting step in LPS biosynthesis, are critical for determining a balanced biosynthesis of LPS and that of phospholipids [9,17,20,38]. LpxC is intrinsically unstable protein and its levels are tightly controlled by its turnover by two essential proteins: FtsH and LapB [17,20,23]. However, it is not known how LapB coordinates with FtsH in this process and if any additional protease is also involved in proteolytic control of LpxC. LapB and FtsH essentiality have been attributed to their role in regulating LpxC turnover. Consistent with such a notion, a deletion of either of these genes can be tolerated in the presence of hyperactive allele of the *fabZ* gene (*sfhC21*) that compensates by diverting common *R*-3-hydroxymyristoyl-ACP to phospholipid biosynthesis [7,17,20]. Alternatively, genetic backgrounds such as *lpxA101* or a Ts mutation in the *lpxD* gene or in genes whose products are required in the early steps of LPS core biosynthesis that reduce LPS biosynthesis or prevent the accumulation of LPS in the IM can rescue the deletion of *lapB* or *ftsH* genes [17,20]. A deletion of the *lapB* gene is lethal in most of the cases, however in certain backgrounds, like BW25113, the Δ*lapB* mutation can be poorly tolerated only on minimal medium at 30 °C but not on rich medium at any temperature. To gain further knowledge about the regulation of LpxC proteolysis and how LapB participates in this process, we employed multi-pronged approaches to address these issues. Firstly, multicopy suppressors were isolated that allow deletion of either *lapB* or *lapA/B* genes and restore growth at elevated temperatures of *lapB* mutant bacteria. These experiments, besides identifying previous multicopy suppressors, led to the cloning of the *hslV* gene. The *hslV* gene encodes the catalytic subunit of prokaryotic counterpart of proteosome and together with HslU has been shown to be involved in turnover of several unstable proteins such as RpoH, RcsA, SulA, dampen the elevated heat shock response and enhance the removal of puromycyl peptides [27,29,30]. Our data show that overexpression of the *hslV* gene alone can reduce elevated levels of LpxC and hence allow growth of either Δ*lapB* or Δ*lapA/B* derivatives. As HslU enhances the proteolytic activity of HslV, we also demonstrated in pulse chase experiments that they together synergistically enhance LpxC proteolysis at elevated temperatures. Thus, by 20 min LpxC amounts are barely detectable, under conditions when the synthesis of all host proteins, including FtsH, is blocked by the addition of rifampicin, with the exception of HslUV. Thus, our results for the first time demonstrate in vivo FtsH-independent degradation of LpxC by HslUV. Our conclusions draw support from computational and system biology-based approach that the predicted LpxC degradation could be regulated by an unidentified protease, whose activity is independent of the lipid A disaccharide concentration [55]. Thus, our experiments establish that this speculated proteolytic activity is due to HslUV, also called ClpQY. Consistent with a role of HslUV protease complex in the regulation of LpxC levels, *hslV* mutants were found to be sensitive to CHIR090 antibiotic that inhibits the activity of LpxC.

The identification of HslUV-dependent proteolysis of LpxC is of relevance, particularly at high temperatures, since transcription of cognate genes is highly induced by the heat shock [27,42]. Interestingly, genome wide interaction studies have shown the interaction of LapA with HslV (UniProt). The HslUV-dependent proteolysis may also become more relevant at high temperature, since the LpxC degradation is inversely correlated with the doubling time [56]. It is likely that the HslUV-dependent degradation comes into play mostly at high temperature and that also draws a parallel between degradation of RpoH at high temperature by HslUV, although FtsH is the primary protease under normal growth conditions. These could also be reasons why HslUV are not essential for bacterial growth like FtsH. The HslUV-dependent proteolysis could also constitute a back-up mechanism to fine tune levels of LpxC under stress conditions. In support of synergistic roles of HslUV and FtsH proteases (Δ*ftsH* Δ*hslVU*) exhibit 10-fold more defect in survival as compared to Δ*ftsH* alone in *Pseudomonas aeruginosa* under carbon starvation conditions [57].

In the second approach, suppressor mutation(s) that allow introduction of Δ*lapB* in W3110 strain identified a novel frame-shift mutation in an essential gene *yejM* designated *lapC* upon further characterization of its genetic interactions. Quite interestingly, the *lapC* gene in certain bacteria is transcribed as a part of operon including the *waaC* gene [58]. We had previously shown that unlike BW25113, a Δ*lapB* mutation is not viable in W3110 a strain that has been widely used in elucidating LPS biogenesis and structural analysis in Raetz laboratory and in many other studies of relevance [4,5,13,14,17]. The frame-shift suppressor mutation in the *lapC* gene *lapC377fs* causes truncation within the periplasmic domain of IM-anchored LapC, highlighting a role for its periplasmic domain. Further characterization of LPS of (Δ*lapB lapC377fs*) derivative provided a significant explanation for LapC function in the regulation of LPS amounts. As previously shown, Δ*lapB* derivatives synthesize more LPS (hence toxicity) and accumulate precursor species of LPS that may not be targeted to the OM correctly [17]. Most importantly, mass spectrometry analysis of LPS of (Δ*lapB lapC377fs*) derivative revealed the restoration of normal LPS composition and suppression of accumulation of early intermediates as is observed in the spectra of LPS of strains lacking LapB. These results suggest that LapB and LapC could together regulate LpxC proteolysis to control the LPS accumulation and act in an antagonistic manner.

In another complementary approach, we set up genetic screens to identify mutations that cause LPS defects, induce the RpoE-dependent envelope stress response and render bacteria sensitive to the LpxC inhibitor CHIR090. RpoE is known to specifically respond to the cell envelope stress including defects in LPS, causing activation of promoters recognized by Eσ^E^ [18,59]. Rationale for the additional screen including the sensitivity to CHIR090 was based on its known inhibition of LpxC and mapping of CHIR090 resistant mutations in the *lpxC* gene [38,39]. Combination of these screens identified three mutations in the *lapC* (*yejM*) gene. The *yejM* gene is known to be essential for growth in *E*. *coli* and loss-of-function mutations in this gene exhibit an imbalance in phospholipid and LPS amounts, although the reasons are not fully understood [31,46]. Among three mutants, *lapC190* and *lapC377fs* conferred a tight Ts phenotype, the hypersensitivity to CHIR090 and the inability to grow on MacConkey agar. Of interest is that we had in an independent genetic approach already identified *lapC377fs* as a suppressor of Δ*lapB* lethality. We further characterized *lapC190* and *lapC377fs* mutations as they conferred a tighter phenotype. Examination of LPS of *lapC190* revealed a drastic reduction in LpxC and LPS amounts. To gain further insights in the function of LapC and its interacting partners, Ts^+^ suppressors that could also restore growth on MacConkey agar albeit to different extents were analyzed further. Suppressors of *lapC190* or *lapC377fs* mutants mapped to the promoter region of the *lapA*/*B* operon and within the structural genes of *lapA*, *lapB*, *lpxC* and *ftsH* all resulting into the increased synthesis of LPS as well as the restoration of LpxC amounts. Based on the severe reduction in the amounts of LpxC in *lapC* mutant bacteria, it is suggested that LapC regulates proteolysis of LpxC. All of suppressor mutations that restored growth at high temperatures and on MacConkey agar mapping to the *lpxC* gene were found to produce more LpxC, indicating these variants are resistant to degradation by FtsH. FtsH has been shown to recognize C-terminal residues in LpxC to initiate degradation [51]. Consistent with this pathway, one of the suppressor mutations exhibiting the increased accumulation of LpxC was found to have a frame-shift mutation at the stop codon resulting into the addition of 20 amino acids. Such a LpxC variant would obviously be not recognized by FtsH. Consistent with suppressors mapping to the *lpxC* gene leading to the stabilization of LpxC, the identification of five independent suppressors causing change of Val37 to Gly (4 out of 5 times) support such conclusions. Structural analysis predicts that this V37G substitution is a change from buried to exposed state and can cause structural damage. Not surprisingly, Val37 alteration in *Klebsiella pneumoniae* has been found to render LpxC more stable and resistant to the LpxC inhibitor [52]. Another variant of LpxC R230C as the suppressor of *lapC* mutants was also isolated three times that also resulted in elevated levels of LpxC. During the completion of this work, two independent studies also reported the isolation of suppressors of *lapC* that caused alterations of either V37 or R230 amino acids, respectively [35,36]. It is of interest that we isolated additional variants of LpxC that suppress *lapC190* and *lapC377fs* mutations, which were not reported in other complementary studies mentioned above, providing a broader model of LpxC regulation and help in understanding the function of LapC.

Consistent with our model that LapC, LapB and FtsH work together to regulate LpxC amounts by the controlled proteolysis and ensuring the balanced LPS synthesis as per demand, the vast majority of suppressors of *lapC* mutant bacteria belong to the category that reduce the LapB abundance or activity by either blocking its transcription or preventing its translation as is evident from the identification of IS elements in the *lapA*-coding region or its translational stop codon. It is worth remembering that *lapA* and *lapB* genes are co-transcribed and translation of LapB is coupled to the LapA synthesis as they have overlapping translational initiation and stop codons [17]. Examination of the LapB amount in all such mutants revealed a drastic reduction in the accumulation of LapB in strains with the IS element in the *lapA* gene or that prevent LapB translation. Regarding suppressor mutations within the coding region of the *lapB* gene, all of them are highly conserved residues in various TPR domains. TPR domains are known to mediate protein-protein interactions [60,61,62] and hence LapB derivatives isolated as suppressors of *lapC* mutants could either prevent the interaction with LapC and FtsH or could be improperly folded rendering LapB nonfunctional. Our modeling analysis suggests that A88V substitution in LapB disrupts all side-chain H-bonds. Another residue H181 in TPR5, whose exchange H181R suppresses the *lapC190* mutation and restores LPS levels, is of significance as H181 mutation was shown to be a loss-of-function mutation in earlier studies [25]. Thus, all such *lapB* variants result into its loss of function, since they mimic the phenotype of Δ*lapB* mutants with the synthesis of more LPS and the reduced accumulation of LapB. This supports our model, in which LapC and LapB act antagonistically and LapC could prevent either the interaction of LapB with FtsH or prevent LapB to act as an IM scaffold directing the LpxC degradation by FtsH. A direct proof for the LapB interaction with LapC was provided in this work based on their co-purification in reciprocal experiments supporting the data from genetic interactions described above. Thus, LapB interacts with FtsH, as demonstrated earlier, and with LapC. Consistent with our data of physical interaction of LapB with LapC, a recent finding published during the preparation of this manuscript have shown that LapC tagged with the FLAG epitope can pull-down LapB [32], consistent with our demonstration of LapB and LapC co-purification.

Since most of the suppressors mapping to the *lapA*/*B* operon synthesize very little LapB, we reasoned that LapB could be dispensable in the *lapC* mutant background. This was indeed established by the ability to transduce the *lapA*/*B* deletion into *lapC190* and *lapC377fs* mutant backgrounds. The absence of LapB would increase the LPS synthesis by stabilizing LpxC and compensate for the reduction in LPS levels in *lapC* mutants. Indeed, this could be shown by measuring the LPS content in various suppressor backgrounds revealing the elevation in the LPS accumulation despite the presence of *lapC190* mutation. Based on the same reasoning, we could show that the ectopic expression of LpxC can render LapC non-essential. Taken together, our findings support a model wherein LapC inhibits the LapB/FtsH-mediated proteolysis allowing the accumulation of LpxC and this inhibition could be relieved, when higher levels of the LPS synthesis are not physiologically required.

However, under what circumstances the LapB interaction with LapC is promoted to direct LpxC to degradation or disrupted to enhance the LpxC synthesis remains to be fully understood. It is conceivable that under stress conditions, for example the exposure to high temperature when the *lapC* mRNA is made more, the LapB/FtsH activity is repressed when LapC is more. Supporting our results of increased accumulation of LapC at high temperature are independent proteomic analysis data of total *E*. *coli* proteome from high temperature or other stress conditions [63,64]. In line with such arguments are findings that LpxC is more rapidly degraded at lower temperatures than at higher temperatures correlating it with doubling time of *E*. *coli* [56]. This would allow the synthesis of more LPS under stress conditions. However, if at high temperature the FtsH-dependent proteolysis of LpxC is limiting, then HslVU protease, whose expression is highly induced at the elevated temperature, could rapidly shift this equilibrium back by the rapid removal of excess of LpxC to maintain the balanced synthesis of LPS.

Thus, in summary based on several lines of evidence we show that LapC is involved in regulating the LPS synthesis and together with FtsH and LapB it balances and regulates the LpxC degradation. As a safety mechanism, HslUV proteases prevent unwanted build-up of LpxC and LPS by promoting proteolysis of LpxC, which could be recruited at high temperatures. Furthermore, the unwarranted accumulation of LPS biosynthetic intermediates in the IM or prior to the LPS delivery to the OM via the Lpt system, when the LapB function is limiting, might relieve the inhibitory effect of LapC and restore the LpxC degradation allowing a balance between phospholipid and LPS biosynthesis.

## 4. Materials and Methods

### 4.1. Bacterial Strains, Plasmids and Media

The bacterial strains and plasmids used in this study are described in Table S1. Luria-Bertani (LB) broth, M9 (Difco, Franklin Lakes, NJ, USA) and M9 minimal media were prepared as described [5]. Whenever required, media were supplemented with ampicillin (100 μg/mL), kanamycin (50 μg/mL), tetracycline (10 μg/mL), chloramphenicol (20 μg/mL) and CHIR090 (0.004 or 0.008 μg/mL). The indicator dye 5-bromo-4-chloro-3-indolyl-*β*-D-galactopyranoside (X-Gal) was used at a final concentration of 40 or 60 μg/mL in the agar medium. Lactose-containing MacConkey agar (Difco) was supplemented with appropriate antibiotics when required. Deletion derivatives used in this study of *hslV*, *hslU*, Δ(*hslVU*), Δ*lapB* and Δ(*lapA/B*) have been previously described [17,27]. Δ*lapC190* strain was constructed using λ-Red mediated recombineering [65].

### 4.2. The Identification of Multicopy Suppressors Whose Overexpression Prevents Lethality of ΔlapA/B Bacteria

The genomic library of all predicted ORFs of *E*. *coli* cloned in pCA24N vector was used to transform Δ(*lapA lapB*) strain SR17187 [17]. Transformants were plated under non-permissive growth conditions of LA medium at 37 and 42 °C in the presence of 75 μM IPTG. Bacterial cultures were grown from such suppressing clones and used to retransform SR17187 to verify the suppression. DNA insert of all relevant plasmids that yielded reproducible results was sequenced. To further validate the suppression by overexpression of either the *hslV* gene alone or co-expression of *hslVU*, previously described plasmids were used [27]. For the induction of HslVU proteins, the minimal coding region of the operon was amplified by PCR using the chromosomal DNA from the wild-type BW25113 strain as a template and cloned in expression vectors such as pTTQ18 or pET24b (Table S1).

### 4.3. Identification of the lapC Gene Based on the Isolation of Mutations That Either Induce Transcription from the LPS-Responsive rpoE*P3*-lacZ Fusion or Suppressor a lapB Deletion

Saturated pools of mini-Tn*10* Kan transposon mutants were generated on LA medium at 30 °C as described previously [66]. A bacteriophage P1 was grown on such pools and transduced into strains SR18868 and SR18987 [18] carrying derivatives of the chromosomal single-copy *rpoE*P3-*lacZ* promoter fusion and subjected to chemical mutagenesis. This promoter was chosen as it specifically responds to severe defects in LPS biosynthesis and is also activated by the Rcs two-component system [18]. Colonies that exhibited a Lac up phenotype (deep blue colonies) on X-Gal-supplemented plates were patched at 30 °C. Approximately more than 80,000 colonies were replica plated at 42 °C. Tn*10*-linked point mutations that conferred a concomitant temperature-sensitive phenotype (Ts) unable to grow at 42 °C and were simultaneously up-regulated for the activity of the *rpoE*P3-*lacZ* promoter fusion were retained. To make selection more stringent, Lac up and Ts isolates were streaked on MacConkey agar at 37 °C and on LA agar supplemented with 0.008 μg/mL of the LpxC inhibitor CHIR090 at 30 °C. Only those candidates that exhibited simultaneous Ts phenotype, unable or poor growth on MacConkey agar, sensitive to growth inhibition by the sub-lethal concentration of the CHIR090 inhibitor and increased the activity of *rpoE*P3-*lacZ* promoter fusion were further verified by bacteriophage P1-mediated transductions and measurement of *β*-galactosidase activity of the *rpoE*P3-*lacZ* fusion. To identify the mutated gene, the position of linked Tn*10* insertion was obtained by sequencing of chromosomal DNA using either inverse PCR as described earlier [17] or sub-cloning of Tn*10* from recombinant cosmid clones that complement the mutation causing LPS defects (see below). Tn*10* insertion or linked Tn*10* point mutations mapping to the main biosynthetic *waa* locus [67] or to other known LPS biosynthetic genes were not followed.

In parallel, we sought extragenic suppressors of a Δ*lapB* or Δ*lapA/B* derivative in W3110 strain. We have earlier reported that a Δ*lapB* derivative is not tolerated in W3110 unless an extragenic suppressor mutation is present. Most of those suppressors were found to synthesize deep-rough LPS (WaaC chemotype). Here we repeated several transductions and screened for Δ*lapB* derivatives in W3110 that synthesize normal amounts of LPS based on reactivity with WN1-225-5 antibody. One such suppressor mutation defined a strain SR8348 and was found by sequence analysis to have Δ*lapB lapC377fs* genotype and was further characterized.

### 4.4. Mapping and Complementation of MacConkey-Sensitive, rpoEP3 Hyperactive and Temperature-Sensitive Mutations

To perform complementation and identify mutated gene, a previously described cosmid library in the single-copy vector was used to transform Ts mutants. Complementing cosmids were obtained by the restoration of growth at 42 °C on MacConkey agar. Plasmids that could recombine mini-Tn*10* and restore growth of Ts mutants on MacConkey agar were used for subcloning and DNA sequencing of cloned inserts. Five Tn*10*-linked Ts mutants mapping at 49 min on the genetic map of *E*. *coli* with the *rpoE*P3-*lacZ* up phenotype and unable to grow on MacConkey agar that identified the *lapC* gene were further followed. To identify the mutation, the chromosomal DNA of the *lapC* gene and its adjoining regions was PCR amplified with appropriate oligonucleotides (Table S2) and its DNA sequence analyzed.

### 4.5. The Isolation of Extragenic Suppressors of lapC Mutants and Their Mapping

As *lapC* F349S, *lapC377fs* and *lapC190* bacteria exhibit the Ts phenotype (inability to grow above 42 °C), several independent cultures of each strain were grown in LB at 30 °C and portions of each plated at 43 °C to seek for temperature-resistant colonies. In parallel in a similar manner, suppressors that restore growth on MacConkey agar were selected by directly plating cultures of *lapC* mutant bacteria on such a selective medium. After verification for their growth at non-permissive growth conditions, mutations were marked with mini-Tn*10* Kan and verified that the Tn*10*-linked suppressor mutation breeds true. The abovementioned cosmid library was used to identify cosmid clones that can recombine linked Tn*10* Kan, which were used to group 26 out of 29 suppressors in three complementation groups. This was further substantiated by additional transductions with defined mutations in linked genes. To further validate this approach, chromosomal DNA of all 29 suppressor strains was isolated and used to PCR amplify candidate genes. In the first series of experiments, chromosomal DNA of 29 suppressors served as a template to amplify the coding and adjoining regions covering the *lpxC* gene. Chromosomal DNA isolated from suppressors belonging to other two complementation groups were next used to PCR amplify the *lapA*/*lapB* operon, including the promoter region, and the *ftsH* gene. PCR products were sequenced using appropriate oligonucleotides listed in the Table S2.

### 4.6. Bacterial Growth Analysis and Measurement of β-Galactosidase Activity

For the quantification of bacterial growth, exponentially grown cultures were adjusted to an optical density OD_595_ of 0.1 and ten-fold dilutions were spot tested on agar plates at different temperatures. Five μL of each dilution was spotted and bacterial growth analyzed after incubation for 24 h at indicated temperatures. To measure the *β*-galactosidase activity, isogenic cultures of the wild type and its derivatives with a specific *lapC* mutation carrying *rpoE-lacZ* promoter fusions were grown overnight under permissive growth conditions. Cultures were adjusted to an optical density OD_595_ of 0.05, allowed to grow at 30 °C for another 45 min. Aliquots of cultures were taken after different intervals as indicated and analyzed for *β*-galactosidase activity as described previously [11]. For each assay, three independent cultures were used and average of each were plotted.

### 4.7. LPS Extraction, Mass Spectrometry and Measurement of LPS Levels

An equivalent amount of bacterial cultures grown up to an OD_595_ of 0.5 were harvested by centrifugation. Pellets were resuspended in 1X sample buffer, boiled for 10 min, followed by digestion with Proteinase K to obtain whole cell lysates. Equivalent portions of such whole cell lysates were applied to a 14% SDS-Tricine gel. After the electrophoresis LPS was transferred by Western blotting. Immunoblots were probed for LPS amounts using the WN1-222-5 monoclonal antibody [50] and revealed by chemiluminescence kit from Thermo Scientific (Warsaw, Poland).

Bacterial cultures (400 mL to 1 L) were grown in phosphate-limiting medium, harvested by centrifugation and pellets were lyophilized. For the LPS analysis, lyophilized material was dispersed in water by sonication and resuspended at a concentration of 2 mg/mL and LPS was extracted by the phenol/chloroform/petroleum ether procedure [68]. Electrospray ionization-Fourier transform ion cyclotron (ESI-FT-ICR)-mass spectrometry was performed on intact LPS in the negative ion mode using an APEX QE (Bruker Daltonics, Breman, Germany) equipped with a 7-tesla actively shielded magnet and dual ESI-MALDI. LPS samples were dissolved at a concentration of ∼10 ng/μL and analyzed as described previously [11,69]. Mass spectra were charge deconvoluted, and mass numbers given refer to the monoisotopic peaks.

### 4.8. Immunoblotting to Measure Amounts of LpxC, LapB and FtsH

The isogenic bacterial culture of wild type and its derivatives carrying various extragenic suppressor mutations and as controls Δ*lapA/B*, Δ*ftsH sfhC21* were grown in either LB or M9 minimal medium at 30 °C to an OD_595_ of 0.5. Equivalent amounts of proteins were applied to a 12% SDS-PAGE and proteins transferred by Western blotting. Blots were probed with polyclonal antibodies against LpxC, FtsH and LapB as described previously. Custom made LapB-specific antibodies were made by Cusabio (Wuhan, China) and used at a dilution of 1:5000. The WN1-225 monoclonal antibody [50] was used at a dilution of 1:10,000. Blots were revealed by chemiluminescence kit from Thermo Scientific as per manufacturer’s instructions.

### 4.9. Purification of LapC and LapB

To co-purify LapA and LapB proteins, the minimal coding region of *lapA*/*B* operon was cloned into the low-copy T7 promoter-based pDUET expression vector (Novagen, Warsaw, Poland) with in-frame deca-His tag at the N-terminus of LapA. The expression of *lapA*/*lapB* genes was induced in BL21 (DE3) derivative by the addition of either 100 or 500 μM IPTG at an OD_600_ 0.2 in 1-L culture medium. For the induction of LapC, the cloned gene in the T7 promoter-based pET28b vector was used and the expression induced with the addition of 200 μM IPTG. Cultures were harvested by centrifugation at 12,000 rpm for 30 min. Pellets were resuspended in B-PER reagent (Pierce) containing 50 mM NaH_2_PO_4_, 300 mM NaCl, 10 mM imidazole. This mixture was supplemented with lysozyme to a final concentration of 200 μg/mL, a cocktail of protease inhibitors (Sigma) and 30 units of benzonase (Merck, Poznan, Poland). The mixture was incubated on ice for 45 min with gentle mixing. The lysate was centrifuged at 45,000× *g* for 30 min at 4 °C and pellets containing IM and OM proteins retained. LapA/B and LapC proteins were extracted using 2% octyl-*β*-D-glucoside for solubilization for IM proteins in the presence of PMSF and a cocktail of protease inhibitors. Solubilized IM proteins were applied over nickel-nitrilotriacetic acid beads (Qiagen, Geneva, Switzerland) and Lap proteins eluted essentially as described earlier [17].

### 4.10. RNA Purification and qRT-PCR Analysis

To measure the mRNA abundance at different temperatures, the bacterial culture of wild-type strain BW25113 were grown at 30 °C in LB rich medium to an OD_600_ of 0.1. For the heat shock, aliquots were immediately shifted to prewarmed medium held at 42 °C and incubated for another 15 min. Equivalent amounts of culture samples were collected from cultures grown at 30 °C and after the heat shock of 42 °C, and harvested by centrifugation. Total RNA was extracted by hot phenol extraction as described previously [70]. Purified total RNA was treated RQ1 DNase (Promega, Madison, WI, USA) to remove any genomic DNA and ethanol precipitated. Pellets were resuspended in DEPC-treated water. RNA amounts were quantified and RNA integrity verified by agarose gel electrophoresis. qRT-PCR was used to quantify changes in the *lapC* gene expression before and after the heat shock treatment. Routinely, 2 μg of purified mRNA was converted to cDNA using iScript Reverse Transcription Supermix from Bio-Rad (Warsaw, Poland). qRT-PCR was performed using CFX Connect Real-Time PCR Detection System (Bio-Rad) as described previously [71]. Data were analyzed by Bio-Rad CFX Maestro software.

## Figures and Tables

**Table 1:** Viable strains lacking the essential *lapB* gene can be constructed, when either the catalytic protease subunit of HslV or when HslUV is overproduced.

Number of Transductants with Selection for Kanamycin Resistance on Minimal Medium			
Donor	Recipient		
	BW25113 + vector	BW25113 + p*hslV*^+^	BW25113 + p*hslUV*^+^
P1 SR17187 Δ*lapA/B* Kan^R^	35 Kan^R^ small colonies	1112 Kan^R^	3230 Kan^R^
P1 SR7753 Δ*lapB* Kan^R^	43 Kan^R^ small colonies	1230 Kan^R^	2980 Kan^R^

**Table 2:** Suppressors of *lapC190* and *lapC377fs* mutations map to *lpxC*, *lapA*/*B* and *ftsH* genes.

Gene	Mutation Position	Amounts of Isolates
***lpxC***	V37G (GTC → GGC)	4
	V37L (GTC → CTC)	1
	R230C (CGT → TGT)	3
	K270T (AAA → ACA)	1
	a frame-shift by the deletion of 2 nt TA from the stop codon resulting into the addition of 20 aa at the C-terminus	1
***lapB***	A88V (GCT → GTT) TPR2	2
	R115H (CGT → CAT) TPR3	1
	D124Y (GAC → TAC) TPR3	1
	R125L (CGC → CTC) TPR3	1
	H181R (CAT → CGT) TPR5	1
	H325L (CAC → CTC) TPR9	1
	H325P (CAC → CCC) TPR9	1
***lapA***	IS element after 34 nt	2
	IS element after 103 nt	1
	IS element after -107 nt - 2 nt after P2hs promoter of the *lapA* gene	1
	a frame-shift by the insertion of G after 69 nt (23 aa from LapA wt and 7 aa new followed by the stop codon)	1
	a frame-shift by the deletion of 137 nt C (45 aa from LapA wt and 12 aa new followed by the stop codon)	1
	LapA L8 (TTA → TGA) stop codon	1
***ftsH***	A296V (GCG → GTG) in the SRH domain	1

**Table 3:** The essential *lapB* gene is dispensable in either *lapC190* or *lapC377fs* backgrounds and the *lapC* gene becomes non-essential if LpxC is overproduced. ND denotes not done.

Number of Transductants with Selection for Kanamycin Resistance on Minimal Medium at 30 °C					
Donor	Recipient				
	BW25113	BW25113 *lapC190*	BW25113 *lapC377fs*	BW25113 + p*lpxC*^+^	
				-IPTG	50 μM IPTG
Δ*lapA/B*Kan	33 Kan^R^ small colonies	2930 Kan^R^ normal size	3470 Kan^R^ normal size	ND	ND
Δ*lapB*Kan	36 Kan^R^ small colonies	3154 Kan^R^ normal size	3696 Kan^R^ normal size	ND	ND
Δ*lapC*Kan	None	ND	ND	None	754

## Supplementary Materials

The following are available online at can be found at https://www.mdpi.com/1422-0067/21/23/9088/s1.

## Abbreviations

L-Ara4N	4-Amino-4-deoxy-L-arabinose
CHIR090	*N*-Aroyl-L-threonine hydroxamic acid
ESI	Electrospray ionization
FabZ	*R*-3-Hydroxymyristoyl acyl carrier protein dehydratase
FT-ICR	Fourier transform-ion cyclotron
Kdo	3-Deoxy-*α*-d-*manno*-oct-2-ulosonic acid
LPS	Lipopolysaccharide
LpxC	UDP-3-*O*-(*R*-3-hydroxymyristoyl)-*N*-acetylglucosamine deacetylase
*P*-EtN	Phosphoethanolamine
TPR	Tetratricopeptide repeat
