# Supplementary material for: Regulation of the First Committed Step in Lipopolysaccharide Biosynthesis Catalyzed by LpxC Requires the Essential Protein LapC (YejM) and HslVU Protease
Source: Int J Mol Sci. 2020 Nov 29;21(23):9088. doi: 10.3390/ijms21239088 (PMC7730581; doi:10.3390/ijms21239088)
Supplement: Supplementary file 1 [file ijms-21-09088-s001.pdf]

Regulation of the First Committed Step in Lipopolysaccharide Biosynthesis Catalyzed by LpxC Requires the Essential Protein LapC (YejM) and HslVU Protease

Daria Biernacka, Patrycja Gorzelak, Gracjana Klein* and Satish Raina*

Unit of Bacterial Genetics, Gdansk University of Technology, Narutowicza 11/12, 80-233 Gdansk, Poland
* Correspondence: gracjana.klein@pg.edu.pl (G.K.); satish.raina@pg.edu.pl (S.R.);
Tel.: +48-58-347-2618 (G.K. & S.R.)

Keywords: lipopolysaccharide; LapB; LapC; YejM; LpxC; HslV/U protease; FabZ; RpoE

Supplementary Materials

Table S1. Bacterial strains and plasmids used in this study

Strains/Plasmids	Relevant characteristic	Reference or source
**Strains**		
BW25113	*lacI*^{q} *rrnB*$_{T14}$ Δ*lacZ*$_{WJ16}$ *hsdR514* Δ*araBAD*$_{AH33}$ Δ*rhaBAD*$_{LD78}$	[65]
T7 Express *lysY/I*^{q}	MiniF *lysY fhuA2 lacZ::T7 gene1 [lon] ompT*	NEB
GK1942	BW25113 (pKD46)	[17]
SR7753	BW25113 (*lapB*)<>*aph*	[17]
SR9523	BW25113 (*lapB*)<>*frt*	[17]
SR8348	SR7753 *lapC* *377fs*	This study
SR16087	BW25113 *lapA-lapB*<>*cat*	[17]
SR17187	BW25113 *lapA-lapB*<>*aph*	[17]
SR19796	SR17187+p*hslV*$^+$	This study
SR17432	BW25113 ϕ (*rpoE*P6-*lacZ*)	[17]
SR18868	BW25113 ϕ (*rpoE*P2/P3-*lacZ*)	[18]
SR18987	BW25113 ϕ (*rpoE*P2*-*lacZ* -12 and -24 mut)	[18]
SR19041	SR18987 *lapC* 190 stop	This study
SR19750	SR18987 *lapC* *377fs*	This study
SR22861	SR18987 *lapC* *377fs*	This study
SR22862	SR18987 *lapC* F349S	This study
GK6075	BW25113 Δ*lapC190*	This study
GK6078	GK6075 *lpxC* K270T	This study
GK6093	GK6075 *lpxC* V37G	This study
SR22727	GK6075 *lpxC* R230C	This study
SR22728	GK6075 *lpxC* R230C	This study
SR22729	GK6075 *lpxC* R230C	This study
SR22731	GK6075 *lpxC* V37G	This study
SR22732	GK6075 *lpxC* V37G	This study
SR22738	GK6075 *lpxC* V37L	This study
SR22739	GK6075 *lpxC* V37G	This study
GK6094	GK6075 *lpxC* *fs*306 stop codon	This study
GK6085	GK6075 *lapA* IS after 34 nt	This study

GK6089	GK6075 *lapA fs* 137 nt	This study
GK6090	GK6075 *lapA* IS after 103 nt	This study
GK6097	GK6075 *lapA* L8 to TGA stop codon	This study
SR22734	GK6075 *lapA* IS after 34 nt	This study
SR22736	GK6075 *lapA* FS after 69 nt	This study
SR22737	GK6075 IS at -106 in *lapAB* promoter region	This study
GK6084	GK6075 *lapB* D124Y	This study
GK6087	GK6075 *lapB* R125L	This study
SR22724	GK6075 *lapB* H325P	This study
SR22725	GK6075 *lapB* H325L	This study
SR22726	GK6075 *lapB* A88V	This study
SR22733	GK6075 *lapB* R115H	This study
GK6095	GK6705 *ftsH* A296V	This study
SR22766	BW25113 Δ*hslUV*<>*aph*	This study
SR22776	SR18987 *lapC190*	This study
SR22777	SR17432 *lapC190*	This study
SR22433	SR18987 *lapC377fs*	This study

Plasmids

pET24b	expression vector	Our collection
pET28b	expression vector	Our collection
pCP20	ts replicon with inducible FLP recombinase	[65]
pKD3	*oriR6K*$_g$, *bla*(Amp^R), *kan*, *rgnB*(Ter), *cat*	[65]
pKD13	*oriR6K*$_g$, *bla*(Amp^R), *kan*, *rgnB*(Ter)	[65]
pKD46	*araBp-gam-bet-exo*, *bla*(Amp^R), *repA101*(ts)	[65]
pCA24N	IPTG-inducible expression vector cm^R	[40]
pSR19788	*hslV*$^+$ in pCA24N	This study
pSR19796	*hslVU*$^+$ in pCA24N	This study
JW2176	*lapC*$^+$ in pCA24N	[40]
pSR22821	*hslVU*$^+$ in pET24b	This study
pSR22901	*lapC*$^+$ in pET28b	This study

Table S2. Primers

For gene disruption:

lapC For	5′-CTC TAT CAA CGA AGA CAA AGC GCA CTA AGG GAA ACA GAT AAC AGG TTA TGA TTC CGG GGA TCC GTC GAC CC-3′
lapC Rev	5′-AGA TAT TTC GCT AAC TGA TTT ATA ATT AAT CAG TTA GCG ATA AAA CGC TTT GTA GGC TGG AGC TGC TTC G-3′

For cloning and overexpression of specific genes:

lapC For	5′-CCG CAT CCC ATG GCT AAA ATT AAA CAT CAC CAT CAC CAT CAC CAC CAT CAC CAT ATG GTA ACT CAT CGT CAG C-3′
lapC Rev	5′-GCA AGT AAG AGA ATT CGC TAA CTG-3′
hslV For	5′-AGG GGT CAG CAT ATG ACA ACT ATA GTA AG-3′
hslU Rev	5′-GAT TGA ACG CTC GAG TCA TTA ATG ATG ATG GTG ATG ATG TAG GAT AAA ACG GCT CAG-3′

For sequencing and PCR amplification:

lapC For	5′-GCG CCT TAC AGT CCT CTA TCA AC-3′
lapC Rev	5′-CGT TTT CCA CAC CGA TTG CAA G-3′
lapA For	5′-GTT GAT TTC GTG GGC GCT GGT G-3′
lapB Rev	5′-GTC GTT GCC GGA GCA CAA TGG-3′
ftsH For	5′-GGA TAT AGA GTA TCC TGA CGC-3′
ftsH Rev	5′-CGG TAC AAA TAC AGT CAT CTG-3′

For q-RT-PCR:

q*lapC* For	5′-ATT CGC AAG GTC AGG ATT TG-3′
q*lapC* Rev	5′-GCG ATA AAA CGC TTC TCG TC-3′
